# 4D Printing of TBCHA-Based Shape Memory Polymer Composites: Bioglass 45S5 Addition Improves Stability Retention and Shape Memory Performance

**DOI:** 10.1021/acsomega.6c00690

**Published:** 2026-04-29

**Authors:** Meng-Ruei Liu, Hsuan Chen, Nien-Ti Tsou, Shyh-Yuan Lee, Yuan-Min Lin

**Affiliations:** † Department of Materials Science and Engineering, 34914 National Yang Ming Chiao Tung University, Hsinchu 30010, Taiwan; ‡ Department of Dentistry, National Yang Ming Chiao Tung University, Taipei 112304, Taiwan; § Institute of Oral Tissue Engineering and Biomaterials, National Yang Ming Chiao Tung University, Taipei 112304, Taiwan

## Abstract

Shape memory polymer composites (SMPCs) were developed by incorporating Bioglass 45S5 into a polycaprolactone diacrylate (PCL-DA)/4-*tert*-butylcyclohexyl acrylate (TBCHA) network to enhance the mechanical robustness, thermal stability, and bioactivity. TBCHA, a bulky acrylate featuring a cyclohexyl-mediated steric architecture, provides a balanced rigid-flexible microstructure that improves interfacial confinement and shape-memory response. Melt-quenched Bioglass 45S5 was uniformly dispersed within the SMP matrix, and the resulting SMPCs were characterized by DSC, DMA, flexural testing, hardness, degradation in PBS, and SBF-induced hydroxyapatite formation. For the SMP30 (lower TBCHA content) network containing 3 wt % Bioglass (SMP 30 BG 3), the glass transition temperature increased from 40 to 45 °C, while the flexural strength increased from 4.3 to 17.46 MPa (≈4.06-fold), together with improved surface hardness. A composition-dependent degradation trend was observed: 1 wt % accelerated degradation, whereas ≥2 wt % slowed it. Even after 2 weeks of accelerated degradation, the 3 wt % composite retained excellent shape-memory performance, with Rf reaching 98.05% (≈3.43-fold that of the degraded neat SMP30), while Rr remained ∼98%. Manual cyclic shape-memory testing over five consecutive thermomechanical cycles further confirmed good cyclic stability, with both Rf and Rr remaining ∼97% after the fifth cycle for SMP 30 BG 3. The steric confinement effect of TBCHA was quantitatively analyzed to elucidate filler–matrix reinforcement. These results demonstrate tunable properties in 4D printable degradable SMPCs with enhanced bioactivity.

## Graphical abstract

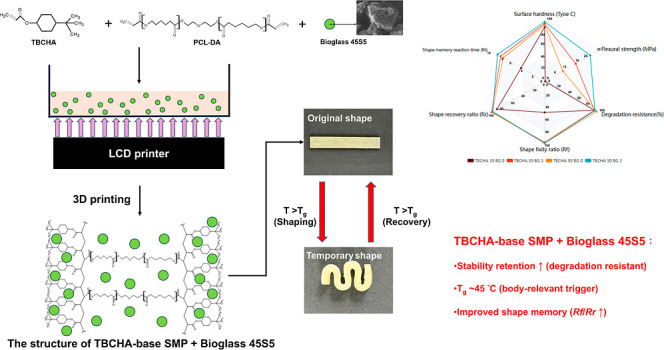

## 1 Introduction

Bone defects and related periodontal-support conditions require biomaterials that can provide temporary structural support, conform to irregular defect geometries, and gradually degrade after healing, thereby reducing the need for secondary removal procedures.^1^ For biomedical use, such materials should also degrade into biocompatible and nontoxic byproducts. Among biodegradable polymers, poly­(ε-caprolactone) (PCL)-based materials have attracted considerable interest because of their biocompatibility, biodegradability, and favorable processability.^1–3^ However, PCL-based systems generally exhibit limited bioactivity and insufficient direct interaction with mineralized tissues. To address this limitation, bioactive ceramic fillers such as Bioglass have been incorporated into polymer matrices to improve apatite-forming ability, osteoconductive potential, and overall biological functionality.^1–6^ Nevertheless, when fabricated as conventional static structures, such materials still show limited adaptability to complex defect shapes or confined implantation sites. In this context, biodegradable 4D printable materials are of growing interest because they combine bioactive composition with the ability to undergo programmed postfabrication shape transformation, which is attractive for minimally invasive deployment and defect-conformal fitting.^7–10^

Among various smart materials, shape memory polymers (SMPs) are especially attractive for such applications because of their tunable transition temperature, programmable deformation, and broad compatibility with additive manufacturing processes.^11^ These features make 4D printed SMPs promising candidates for tissue engineering, regenerative medicine, and implantable biomedical devices. However, integrating bioactive fillers into biodegradable SMP systems remains challenging. Although Bioglass can improve bioactivity and osteoconductive potential, previous studies on SMPCs and filler-containing systems have shown that particle aggregation, pore-wall localization, and restricted thermal-processing windows may compromise mechanical integrity or controllability of the shape-memory response.^12–15^ These limitations indicate that the performance of biodegradable bioactive SMPCs depends not only on filler loading but also on matrix chemistry, which governs the stability of the filler–matrix interfacial region and the chain mobility required for shape-memory behavior. To tailor the thermal and mechanical responses of SMPs, diverse molecular and compositional strategies have been explored. Conventional acrylate-based systems often combine soft and hard segments, such as poly­(ε-caprolactone) diacrylate (PCL-DA), poly­(ethylene glycol)­diacrylate (PEGDA), and urethane acrylates, to achieve elasticity, cross-linking control, and processability suitable for 4D printing.^11^ In addition, blending strategies and functional fillers have been widely used to modulate shape memory behavior and broaden the functional window of 4D printed SMPs.^8–10^ However, most existing approaches rely primarily on polymer blending, plasticization, or filler incorporation, whereas comparatively less attention has been paid to monomer-level structural design as a route to regulate chain mobility, rigidity, and filler–matrix compatibility in biodegradable photocurable networks. For biodegradable bioactive SMPs, such molecular-level design is particularly important because the polymer network must not only accommodate the ceramic filler but also stabilize the filler–matrix interfacial region while preserving the segmental mobility required for effective shape fixation and recovery, especially after degradation.

In this context, *tert*-butylcyclohexyl acrylate (TBCHA) offers a useful steric design platform. Common bulky or shape-memory-relevant acrylate monomers, such as *tert*-butyl acrylate (tBA), isobornyl acrylate, and hydroxybutyl acrylate, can contribute to shape fixity and tunable thermal response, but often involve compromises among stiffness, dimensional stability, and recovery behavior.^16–18^ In our earlier study, TBCHA was introduced as an alternative to tBA to overcome the latter’s relatively flexible structure and limited dimensional stability during fixation.^19^ Compared with tBA, TBCHA contains an additional cyclohexyl ring between the acrylate group and the *tert*-butyl substituent, giving rise to a more sterically demanding and conformationally complex architecture. This structural feature is expected to increase local steric hindrance and dimensional stability while retaining conformational adaptability for shape recovery through the nonplanar cyclohexyl chair structure.^20^ Therefore, unlike conventional bulky acrylates that are often selected mainly to raise the glass transition temperature (*T*_g_) or stiffness, TBCHA may offer a distinctive design advantage by introducing a sterically regulated rigid-flexible microenvironment that reinforces mechanical integrity while preserving the segmental mobility necessary for shape recovery. More importantly, TBCHA is considered here not merely as an alternative bulky acrylate but also as a sterically regulated monomeric design element for controlling the microenvironment around Bioglass particles in a degradable shape-memory network. Owing to its bulky cyclohexyl ring and *tert*-butyl substituent, TBCHA may regulate local chain packing and interfacial confinement around the filler,^20^ thereby stabilizing the filler–matrix interfacial region at the molecular-chain level. Such steric regulation may help the surrounding polymer network accommodate Bioglass within a mechanically coherent matrix, reduce filler-induced disruption of cooperative segmental motion, and preserve network integrity during degradation. As a result, TBCHA may support the retention of strong shape-memory performance, even after degradation.

Based on this rationale, the present study investigates biodegradable PCL-DA/TBCHA-based SMPCs containing different amounts of Bioglass 45S5 to achieve a balance among mechanical stability, shape memory behavior, degradation response, and bioactivity. Unlike many previous studies that mainly emphasize qualitative actuation or initial recovery behavior, this work further evaluates shape fixity and shape recovery after degradation and interprets the observed thermal and mechanical behavior from the perspective of polymer–filler interfacial interactions. More importantly, this study treats TBCHA not merely as an alternative bulky acrylate but also as a sterically regulated monomeric design strategy for stabilizing the polymer–filler interfacial microenvironment in biodegradable, bioactive, and 4D printable SMP composites, thereby helping preserve shape-memory functionality after degradation for bone and periodontal tissue regenerative applications.

## 2 Materials and Methods

### 2.1 Materials Synthesis

#### 2.1.1 Melt-Quenching Method to Synthesize the Bioglass 45S5

Figure 1a illustrates the synthesis process flowchart for Bioglass 45S5. In this case, Bioglass 45S5 was synthesized by the melt-quenching method with the following composition: SiO_2_: 45 wt %, CaO: 24.5 wt %, Na_2_O: 24.5 wt %, and P_2_O_5_: 6 wt %. The powdered raw materials were thoroughly mixed and placed in an alumina crucible and then melted at 1400 °C for 1 h under an inert atmosphere. The homogenized melt was then quenched in cold water to rapidly solidify the glass. To release internal stresses generated during the rapid cooling process, the vitrified glass was annealed at 520 °C for 1 h and then allowed to cool gradually to room temperature. The melt-quenched Bioglass 45S5 was manually ground and sieved by using a two-sieve setup. Particles passing through a 200 mesh sieve and remaining on a 400 mesh sieve were collected. The particle size distribution of this fraction was further quantified from SEM micrographs using ImageJ. Briefly, the ImageJ scale was calibrated using the scale bar in each image, and individual particles were manually measured to determine their characteristic dimensions, while overlapped or severely agglomerated regions were excluded. The measured particle size distribution is presented in the main text, and the detailed analysis procedure is provided in Supporting Information Section 1.5 Particle size analysis. Based on the sieve fraction and SEM/ImageJ analysis, the collected particles were in the range of approximately 38–75 μm and were used for composite preparation.

**Figure 1:**
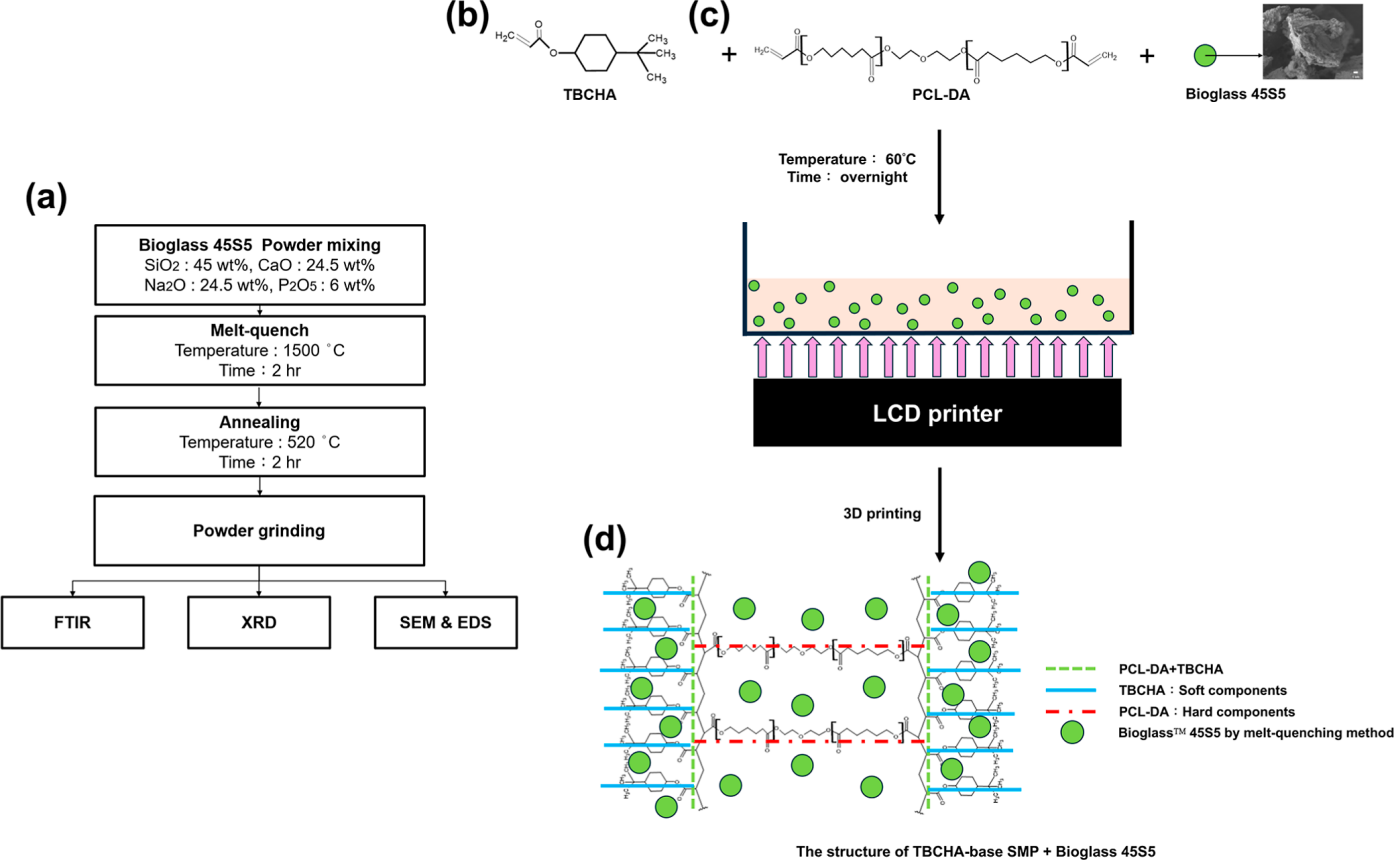
(a) The synthesis process flowchart for Bioglass 45S5; (b) the TBCHA is the soft component monomer of SMP; (c) PCL-DA is the hard component monomer of SMP, and (d) the structure of the SMPC.

#### 2.1.2 Synthesis of Polycaprolactone Diacrylate

Polycaprolactone diol (PCL-Diol, Mn = 2000, Scientific Polymer Products Inc., USA) was dissolved in toluene (99%, Echo Chemical Co., Ltd., Taiwan) to achieve a solution with a 35 wt % concentration of PCL-Diol at room temperature. Once fully dissolved, *p*-toluenesulfonic acid monohydrate (*p*-TSA, 99%, extra pure, ACROS ORGANICS, USA) was added as a catalyst, and *tert*-butylhydroquinone (TBHQ, Mw = 166.22, Tokyo Chemical Industry Co., Tokyo) was introduced as an inhibitor to prevent unwanted side reactions. Acrylic acid (AA, 98%, extra pure, Thermo Scientific, USA) was then added to the solution in a molar ratio of PCL-Diol to AA of 1:2. The resulting reaction mixture was stirred continuously using a magnetic stirrer (PC-420D, Corning, USA) at 60 °C for 24 h to facilitate the esterification process. After the reaction, the mixture was precipitated into cold *n*-hexane (95%, Seedchem Company, Australia). The upper solvent layer was carefully decanted, and fresh room-temperature *n*-hexane was added to the precipitate. The mixture was stirred at 1000 rpm for 15 min at room temperature. Subsequently, the solution was transferred to a refrigerated environment at 4 °C to allow for further precipitation of the PCL-DA product. This washing process was repeated at least three times to ensure the thorough removal of residual acrylic acid and byproducts.

The purified PCL-DA was then dried in a vacuum drying oven (IACF-DOV30, DENG YNG, Taiwan) at 40 °C and 60 cmHg until a constant weight was achieved, confirming the complete removal of residual solvents. The final product was obtained as a snow-white wax and stored for subsequent characterization and experimental applications.

#### 2.1.3 Synthesis of SMPC 4D Print Resin

Figure 1b–d shows the synthesis process of SMP in this study. The synthesized PCL-DA was combined with TBCHA (Mw = 210.31, Miwon Specialty Chemical Co., South Korea) at a predetermined molar ratio. To improve printing precision and reduce printing time, (−)-Riboflavin (from Eremothecium ashbyii, ≥98%, R4500, Sigma-Aldrich, USA) was incorporated as a photoabsorber. Diphenyl­(2,4,6-trimethylbenzoyl) phosphine oxide (TPO) (DOUBLECURE TPO, Double Bond Chemical Co., Taiwan) was then added as the photoinitiator to facilitate the photopolymerization process. Additionally, Bioglass 45S5, synthesized by using the melt-quenching method, was introduced into the formulation to confer osteogenic bioactivity to the resin.

The resin mixture was thoroughly stirred at 60 °C overnight using a magnetic stirrer to ensure uniform dispersion of the Bioglass 45S5 particles, the photoinitiator, and the photoabsorber. This resulted in a homogeneously mixed liquid resin. To minimize gravitational settling of the micrometer-sized particles, the suspension was printed within a short time window after mixing. After mixing, the filled resin was allowed to stand for ∼10 min to release entrapped air bubbles before printing; if necessary, the resin in the vat was gently stirred immediately prior to printing to restore homogeneity. The final resin formulation was employed in LCD-based photopolymerization and 3D printing for material fabrication.

The dispersion state of Bioglass 45S5 within the printed composites was further examined using SEM fracture cross sections (Supporting Information Section 1.1 SEM/EDS cross-sectional analysis of Bioglass 45S5 dispersion in printed composites). No obvious large agglomerates or sedimentation-induced concentration gradients were observed within the inspected cross-sectional regions.

### 2.2 Printing Process

The detailed printing parameters are summarized in Table 1. The exposure time was selected based on a single-layer cure-depth screening conducted using TBCHA 30 BG 3 and TBCHA 50 BG 3, which represented the worst-case formulations. Detailed results of the curing tests are provided in the Supporting Information (Section 1.2, Printing parameters test). For TBCHA 30 BG 3, the measured cure depths were 0.04, 0.07, 0.12, and 0.25 mm at exposure times of 15, 30, 45, and 60 s, respectively. An exposure time of 45 s was chosen because the corresponding cure depth (∼0.12 mm) slightly exceeded the layer thickness (0.10 mm), thereby ensuring sufficient interlayer bonding without substantial overcuring. A comparable trend was also observed for TBCHA 50 BG 3 (Supporting Information Section 1.2 Printing parameters test). The samples and supporting structures were initially designed using an Autodesk Fusion 360 (Autodesk Tinkercad, Autodesk Inc., USA). The finalized designs were exported as.stl files and imported into CHITUBOX (Chitubox, China) for preprint processing. The models were then sliced with a layer thickness of 100 μm. Following each printing process, the printed samples were cleaned using an ultrasonic cleaner (Formwash, Formlabs Co., USA) for 15 min to remove residual resin thoroughly. A 95% ethanol solution (Delight Ethanol Co., Taoyuan City, Taiwan) was used during the cleaning procedure. Subsequently, the samples were transferred to a postcuring chamber (Form Cure, Formlabs Co., USA) and subjected to postcuring at 60 °C for 15 min to finalize the polymerization process. Table 2 illustrates the proportions of TBCHA and Bioglass 45S5 used in the SMP samples for this study.

**Table 1:** Printing parameters

layer height	100 μm
bottom layer count	5
transition layer count	20
transition type	linear
exposure time	45 s
bottom exposure time	60 s
light-off delay	7 s
lifting distance	6 mm
lifting speed	65 mm/min
retract speed	150 mm/min

**Table 2:** Sample Name of All Groups

sample name	TBCHA	Bioglass 45S5 (BG)
TBCHA10 BG 0	TBCHA 10	0 wt %
TBCHA10 BG 1		1 wt %
TBCHA10 BG 2	(TBCHA; 49.56 wt %)	2 wt %
TBCHA10 BG 3		3 wt %
TBCHA30 BG 0	TBCHA 30	0 wt %
TBCHA30 BG 1		1 wt %
TBCHA30 BG 2	(TBCHA; 74.67 wt %)	2 wt %
TBCHA30 BG 3		3 wt %
TBCHA50 BG 0	TBCHA 50	0 wt %
TBCHA50 BG 1		1 wt %
TBCHA50 BG 2	(TBCHA; 83.09 wt %)	2 wt %
TBCHA50 BG 3		3 wt %

### 2.3 Fourier Transform Infrared Spectroscopic Analysis

Fourier transform infrared (FTIR) spectroscopy (Nicolet iS5, Thermo Fisher Scientific, USA) equipped with a ZnSe ATR crystal was employed to investigate possible interfacial interactions between the polymer matrix and Bioglass 45S5. Spectra were collected in the range of 2000 to 650 cm^–1^ at a spectral resolution of 4 cm^–1^, averaging 16 scans per sample (background acquired prior to each measurement). Samples were measured in direct contact with the ATR crystal under a constant pressure. The analysis focused on characteristic absorption bands corresponding to C=O stretching (∼1720 cm^–1^) and Si–O–Si/Si–O–Ca vibrations (∼1100–1000 cm^–1^) arising from the incorporation of Bioglass. Shifts or intensity variations in these regions were used to assess potential hydrogen bonding or ionic coordination between polymer carbonyl groups and Bioglass surface sites.

### 2.4 Thermal Property Characterization (DSC and DMA)

The thermal properties of the SMPs were analyzed using differential scanning calorimetry (DSC 4000, PerkinElmer, USA) under a nitrogen purge (20 mL/min), a widely utilized technique for investigating the thermal behavior of polymers. The first cycle aimed to eliminate residual solvents and impurities from the samples, thereby enhancing the accuracy and reliability of the subsequent measurements. By minimizing potential interferences, the second heating cycle provided precise thermal data. The samples were heated from 0 to 100 °C at 10 °C/min during the first heating cycle. The samples were rapidly cooled to −20 °C and held at that temperature for 3 min to equilibrate. The second heating cycle then commenced, heating the samples from −20 to 100 °C at a constant rate of 10 °C/min. This cycle was conducted to determine the *T*_g_ value of the SMPC accurately.

DMA provides critical insights into materials’ stiffness and damping properties, quantified as the modulus and tan delta. Subjecting the sample to a sinusoidal force separates the modulus into two components: the storage modulus (*E*′) and the loss modulus (*E*″). The *E*′ reflects the material’s elastic behavior, representing its ability to store and release energy during deformation, whereas the *E*″ indicates the energy dissipated as internal friction within the material. Rectangular specimens with dimensions of 2 × 5 × 40 mm^3^ were used for the DMA tests. The experiments were conducted using a dynamic mechanical analyzer (DMA 850, stress-controlled mode, TA Instruments, USA) in a three-point bending configuration at a frequency of 1 Hz. The temperature range (−50 to 70 °C) was selected based on body temperature and the *T*_g_ determined in a previous study using DSC. To ensure precise measurements, a heating rate of 3 °C/min was employed to minimize potential artifacts induced by rapid temperature changes. Regarding the gas conditions, the instrument was operated with a compressed-air supply (approximately 80 psi upstream and 70 psi after the air filter); no purge gas was applied during the DMA measurement, and the gas flow rate was not controlled by the facility. The raw experimental data and input parameters, including specimen dimensions, deformation mode, and temperature profile, were analyzed by using equations integrated within the TA Instruments software.

### 2.5 Mechanical Property Test

Each formulated resin’s surface hardness was evaluated using a Shore Type C durometer (GS-751G, Teclock Co., Japan) following the ASTM D2240 standard. Specimens were fabricated with dimensions of 10 × 10 × 3 mm^3^. Hardness measurements were performed twice for each of the six sample formulations, and the average values were calculated to ensure reliability and accuracy.

This study measured the three-point bending of each designed resin using a Universal Testing Machine (QC-513B1, Kangtai Testing Machine Co., Ltd., Taiwan) following the ASTM 20795-1 standard. The specimens were prepared as 64 × 10 × 3.3 mm^3^ samples. One measurement was performed on each of the six samples, and the mean values were calculated.

### 2.6 Shape Memory Characterization

The shape memory properties, including Rf, Rr, and Rt, were evaluated using a custom-designed angled mold, a heat gun (SL-1500, SULI Co., Taiwan), and a protractor. The sample was initially heated from 25 to 80 °C, surpassing its *T*_g_, and maintained at this temperature for 5 min. A ramped force was then applied, bending the sample to different angles (0°, 45°, and 90°), followed by cooling to 0 °C while the applied force. After cooling and unloading, the temporary shape was stabilized by holding the specimen at 37 °C for 1 h, after which Rf was recorded. The specimen was subsequently reheated to 80 °C and held for 5 min to evaluate Rr. The shape memory behavior was evaluated by calculating the shape fixity ratio (Rf) and the shape recovery ratio (Rr). The Rf was determined by comparing the difference between the programmed deformation angle (θ_0_) and the fixed angle after unloading (θ) with the programmed angle, reflecting the ability of the material to maintain its temporary shape. The Rr was determined by comparing the recovered angle (θ) with the programmed deformation angle (θ_0_), representing the efficiency of the material in restoring its original shape after reheating. A schematic of the test procedure and the corresponding angle definitions used for eqs 1 and 2 are provided in Figure 2.

1 $$Rf\;\left(\%\right)=\left(\theta_{0}-\theta \right)/\theta_{0}\times 100\%$$

2 $$Rr\;\left(\%\right)=\theta /\theta_{0}\times 100\%$$

**Figure 2:**
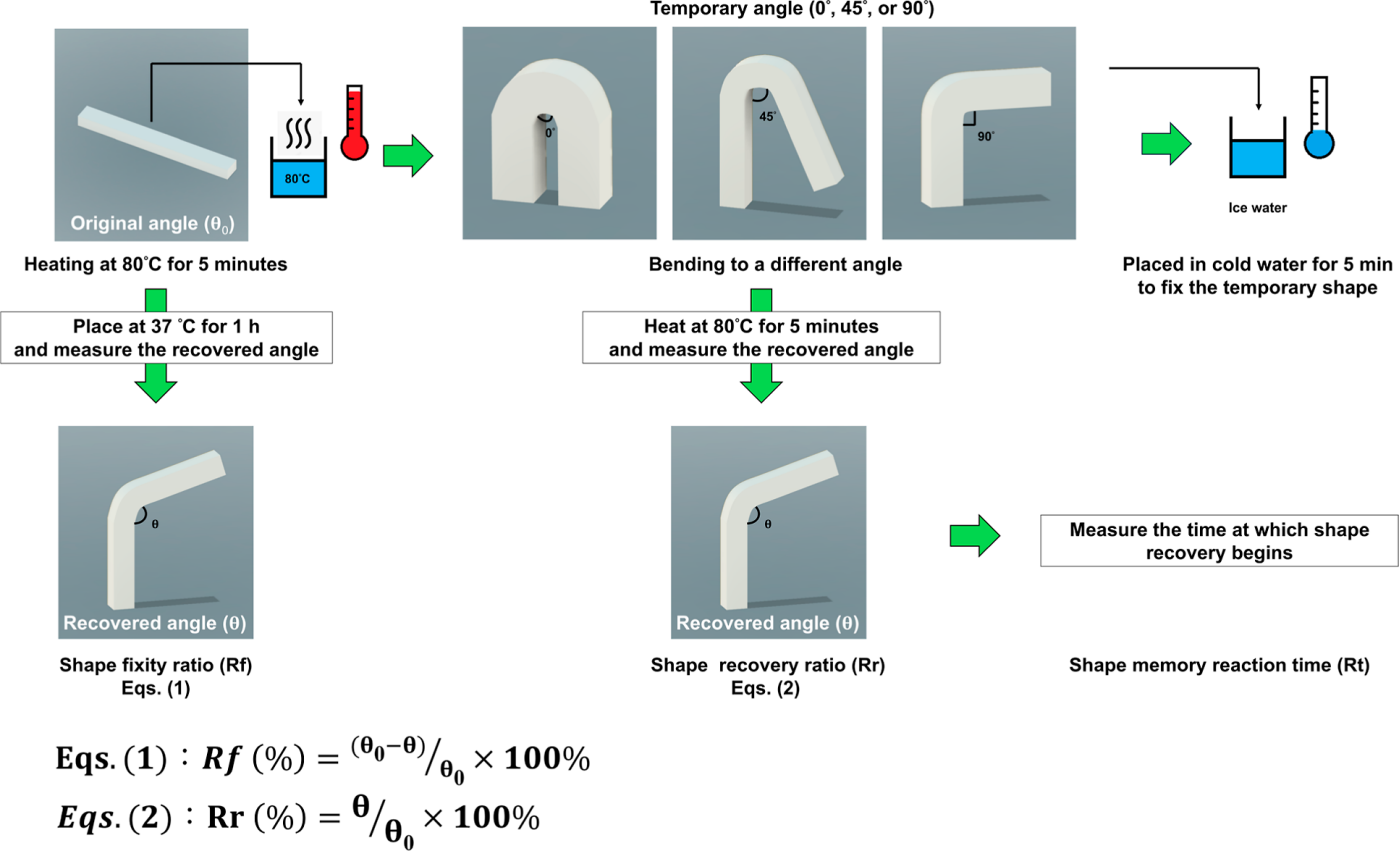
Schematic illustration of the shape-memory testing procedure.

This methodology was used to assess the shape memory behavior of the samples, specifically their ability to fix a deformed shape (Rf) and recover their original shape upon reheating (Rr). The samples were bent to different angles during testing to evaluate their application limitations and to inform future research directions.

For manual cyclic shape-memory testing, Rf and Rr were measured under the same experimental conditions described above, and the procedure was repeated for five consecutive cycles.

### 2.7 Degradation Characterization

Accelerated degradation tests were performed in a highly basic medium to address the extended degradation time of PCL. The specimens, shaped as cuboids with dimensions of 10 × 10 × 2 mm^3^, were pretreated by washing and immersing them in 95% ethanol for 15 min, followed by drying at room temperature for 24 h to achieve a constant weight. The initial weight of each specimen was recorded. The specimens were then immersed in 15 mL tubes containing a 5 M NaOH solution (1000 wt % % relative to the SMP) prepared using PBS. The tubes were incubated in an oven at 37 °C for designated intervals (2, 4, 6, 8, 10, 12, 14, and 16 days). The degradation solution was collected at each time point, and its pH value was measured. The specimens were retrieved, ultrasonically cleaned, and then washed and immersed in deionized water for 30 min. Afterward, they were carefully wiped, weighed, and vacuum-dried to a constant weight. The extent of degradation was evaluated by measuring the weight loss of the SMP specimens. The weight loss percentage was determined by comparing the dry weight after degradation (Wd) with the initial dry weight (Wo) using the eq 3

3 weightloss=(Wo−Wd)/Wo×100%

### 2.8 Shape Memory Characterization after Degradation

Accelerated degradation tests were performed in a highly basic medium to address the extended degradation time of PCL. The specimen’s shape follows the ASTM 20795-1 standard with dimensions of 64 × 10 × 3.3 mm^3^ and was pretreated by washing and immersing it in 95% ethanol for 15 min and then drying at room temperature for 24 h to achieve a constant weight. The specimens were then immersed in 150 mL tubes containing a 5 M NaOH solution (1000 wt % % relative to the SMP) prepared using PBS. The tubes were incubated in an oven at 37 °C for designated intervals (7 and 14 days). The specimens were retrieved, ultrasonically cleaned, and then washed and immersed in deionized water for 30 min. Afterward, they were carefully wiped, weighed, and vacuum-dried to a constant weight. Follow the methodology outlined in Section 2.6. Shape Memory Characterization to conduct the shape memory properties test, including evaluating Rf, Rr, and Rt. Shape-memory properties after degradation were evaluated by following the procedure described in Section 2.6.

### 2.9 In Vitro Bioactivity Characterization

Kokubo SBF is a solution with ion concentrations nearly identical to those of human blood plasma, making it an effective medium for simulating the in vivo conditions of materials within the human body. The preparation of SBF followed Professor Kokubo’s protocol as described in the referenced study.^21^ To evaluate bioactivity, the samples were immersed in SBF for 28 days; the immersion medium was replaced every 2 days. After immersion, the surface morphology and composition were analyzed by scanning electron microscopy (FE-SEM, JEOL JSM-7600F, Japan), energy-dispersive X-ray spectroscopy (EDS), and X-ray diffraction (XRD, Bruker D8-ADVANCE, USA). As a positive control for bioactivity, pristine Bioglass 45S5 was also immersed in SBF under identical conditions; the results are provided in Supporting Information, Section 1.3 (In vitro bioactivity of Bioglass 45S5), and discussed in Section 3.8 (In vitro bioactivity of TBCHA-based SMPC).

### 2.10 Statistical analysis

Multiple samples were used for various experimental analyses, and the data obtained from experiments were reported as the mean ± standard deviation. For most tests, the number of replicates was *n* = 6, except for the degradation and in vitro bioactivity tests, where *n* = 3. Statistical comparisons between groups were performed using one-way analysis of variance (ANOVA). Statistical significance was defined as *p* < 0.05. In addition, the coefficient of determination (*R* ^2^) was calculated for each one-way ANOVA model, reported in the corresponding figure captions, and summarized in detail in the Supporting Information [Section 1.4, Statistical analysis (ANOVA *R* ^2^ values)].

## 3 Results and Discussion

### 3.1 Structural, Morphological, and Chemical Characterization of Bioglass 45S5

Figure 3 illustrates Bioglass 45S5 synthesized by the melt-quenching method, which was analyzed using SEM. Figure 3a indicates that Bioglass 45S5 obtained through the melt-quenching method exhibited an irregular powder morphology. Figure 3b shows that EDS analysis confirmed the presence of Calcium (Ca), Phosphorus (P), Sodium (Na), and Silicon (Si) peaks. Additionally, the figure presents the results of the EDS mapping mode, which demonstrated that these elements within the inspected particles and fields of view indicate a chemically uniform glass composition. Figure 3c presents the particle size distribution of melt-quenched Bioglass 45S5. The particle size analysis was conducted using ImageJ based on SEM images of the melt-quenched Bioglass 45S5 (Supporting Information, Section 1.5 particle size analysis). The results indicated that the particle size was mainly distributed between 30 and 70 μm, which is consistent with the sieved fraction of 200 to 400 mesh (38 to 75 μm). Figure 3d illustrates the FTIR analysis result of Bioglass 45S5 synthesized by the melt-quenching method. The FTIR spectra revealed characteristic peaks, including a Si–O–Si peak in the range of 1030 to 1090 cm^–1^, a Si–O peak at 980 cm^–1^, a P–O peak in the range of 600 to 800 cm^–1^, and a CO_3_^2–^ peak in the range of 1400 to 1500 cm^–1^. The characteristic FTIR features observed in this study indicate that Bioglass 45S5 was successfully synthesized via the melt-quenching method. Figure 3e illustrates the XRD analysis result of Bioglass 45S5 synthesized by the melt-quenching method. The XRD pattern indicates the absence of sharp diffraction peaks. Instead, a broad peak, commonly called an amorphous halo, is observed around 25 to 30° (2θ).^22^ This confirms that Bioglass 45S5 prepared using the melt-quenching method exhibits an amorphous structure. According to the literature, amorphous Bioglass 45S5 demonstrates excellent bioactivity by reacting with body fluids to form the HA layer.^22^ The similarity between these results further validates that the melt-quenching method successfully produces amorphous Bioglass 45S5.

**Figure 3:**
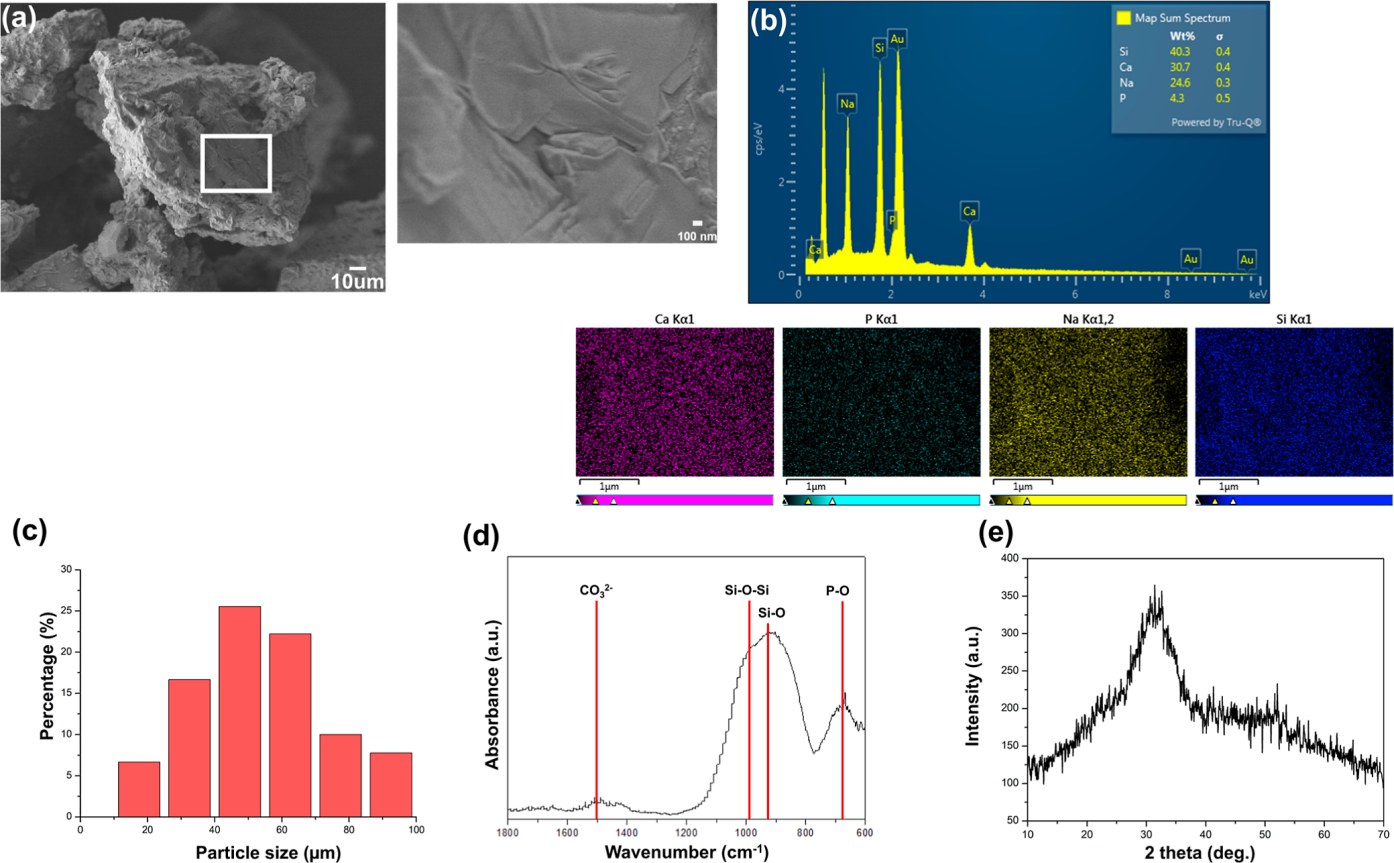
(a) Bioglass 45S5 prepared via melt-quenching; (b) the morphology and composition of Bioglass 45S5 (magnification (main/inset): ×5000/×35,000); (c) the particle size distribution of melt-quenched Bioglass 45S5; (d) the FTIR characteristic peaks of Bioglass 45S5 synthesized by the melt-quenching method; (e) the XRD characteristic peaks of Bioglass 45S5 are synthesized by the melt-quenching method.

### 3.2 FTIR Analysis of TBCHA-Based SMPC Resin

Figure 4 illustrates the FTIR spectra of all of the TBCHA-based SMPC resins. All samples exhibit the characteristic ester C=O stretching near 1720 cm^–1^. With increasing Bioglass 45S5 content, a distinct enhancement appears around 1150 cm^–1^ only in the TBCHA 10 series, whereas the TBCHA 30 and 50 series remain nearly unchanged. The ∼1150 cm^–1^ band arises from the overlapping C–O–C stretching of the polymer and Si–O–Si asymmetric stretching of Bioglass, indicating noncovalent interfacial interaction between Bioglass and the PCL-DA-rich matrix.^23^ As the TBCHA content increases, the bulky, nonpolar cyclohexyl and *tert*-butyl substituents sterically shield adjacent carbonyl and ether groups, limiting hydrogen bonding or Ca^2+^ coordination at the filler–matrix interface.^20^ Consequently, the interfacial interaction becomes predominantly physical rather than chemical, with the cyclohexyl structure modulating the extent of Bioglass–polymer coupling, being strongest in TBCHA 10 and progressively diminishing in TBCHA 30 and 50.

**Figure 4:**
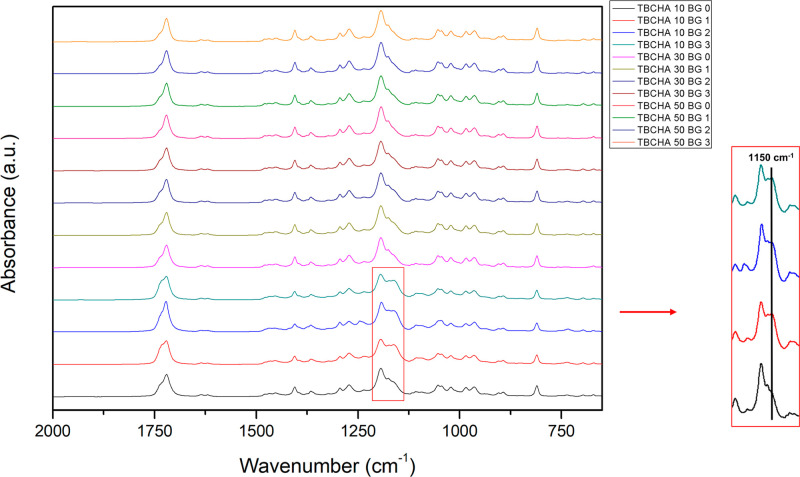
FTIR spectra of all TBCHA-based SMPC resins.

### 3.3 Thermal Analysis of TBCHA-Based SMPC

The driving force behind the shape memory properties of SMPs is the temperature exceeding the *T*_g_, which triggers the transformation of SMPs from a glassy state to a rubbery state.^24^ Considering that changes in cross-link density and the addition of particulate fillers may restrict the free movement of molecular chain segments,^25^ the design of materials for biomedical implants requires a *T*_g_ within an appropriate range. If the *T*_g_ is excessively high or low, then the material may fail to meet the requirements for biomedical implant applications. Therefore, DSC was performed to evaluate the *T*_g_ values of the SMPC samples.

Figure 5a,b illustrates the results for the TBCHA 30 and TBCHA 50 samples. For the TBCHA 10 group, thermal analysis was not performed because the TBCHA 10 BG 0 exhibited a *T*_g_ below room temperature and did not display shape memory behavior, while the Bioglass-containing samples were mechanically too soft to yield meaningful results. The *T*_g_ was relatively low for TBCHA 30 BG 0, approaching room temperature. This indicates that the sample transitions from a glassy state to a rubbery state at room temperature, which causes the implant to recover its original shape before implantation, which is undesirable. However, with increasing Bioglass 45S5 content, *T*_g_ exhibited an upward trend. This is primarily because the addition of particulate fillers restricts the free movement of molecular chain segments, enhancing structural stability and increasing the *T*_g_.^25^ In contrast, the TBCHA 50 group demonstrated a higher *T*_g_. Furthermore, the flatter curve observed in this group indicates reduced chain crystallization within the polymer, attributed to an increased cross-link density.^26^ This phenomenon can be explained by more intermolecular connections, which restrict the polymer chain mobility and hinder the formation of crystalline regions. Conversely, an increase in the added Bioglass 45S5 content within the same group leads to a more pronounced bending curve, suggesting an enhancement in the crystallization. These results indicate that a higher TBCHA monomer content leads to increased cross-link density to enhance thermal stability, and higher Bioglass 45S5 content can also improve the material’s thermal stability. Notably, the *T*_g_ values measured via DSC for TBCHA 30 BG 3 and TBCHA 50 BG 3 were approximately 45 °C, within the clinically acceptable range for in vivo shape recovery applications. A *T*_g_ in this range allows thermal triggering through mild local heating (e.g., warm saline or infrared stimulation), avoiding the risk of tissue damage associated with higher temperatures. Therefore, the observed *T*_g_ supports the potential for safe and effective biomedical use of these SMPC.

**Figure 5:**
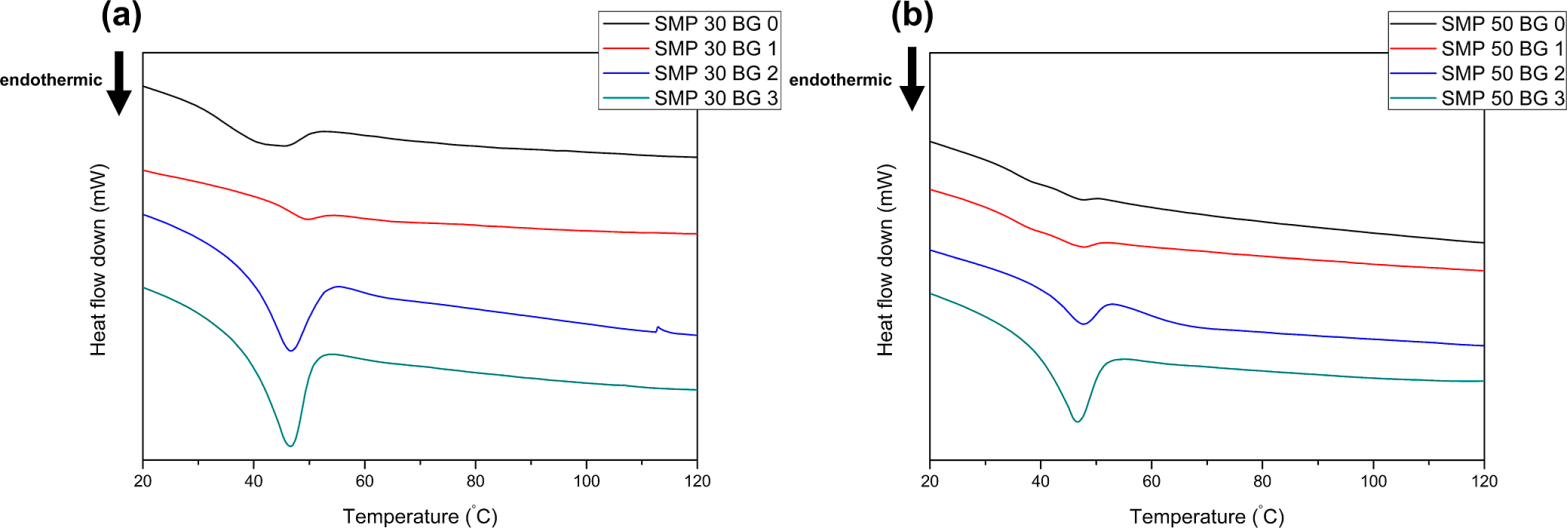
(a) The DSC curves of the TBCHA 30 sample and (b) the TBCHA 50 sample.

DMA is a versatile technique for evaluating the mechanical properties of materials. Among the key parameters obtained from DMA is the tan delta, also called the loss factor. Tan delta represents the ratio of energy dissipated as heat (*E*″) to energy stored elastically (*E*′) within the material.^27^ In SMPCs, the tan delta plays a significant role in shape recovery and fixation efficiency. A sharp and distinct tan delta peak near *T*_g_ facilitates rapid energy dissipation during recovery, ensuring reliable and efficient shape memory behavior.^28^ Moreover, the magnitude of the tan delta influences the suitability of materials for specific biomedical applications: low tan delta indicates a reduced energy dissipation and enhanced elasticity. Such materials are desirable for structural applications requiring minimal deformation under load, such as bone implants and load-bearing scaffolds. High tan delta reflects higher energy dissipation capabilities, making these materials more appropriate for vibration-damping or shock absorption applications, such as cartilage replacements, soft tissue implants, and cushioning layers.^29^

The *E*′ represents the material’s ability to store elastic energy and is a key indicator of stiffness. As illustrated in Figure 6a–d, the *E*′ values for the non-Bioglass 45S5 groups TBCHA 50 BG 0 are higher than TBCHA 30 BG 0, reflecting the increased cross-link density due to the higher TBCHA content. Moreover, incorporating 3 wt % Bioglass 45S5 further increased *E*′, particularly in the TBCHA 50 BG 3 sample. This suggests that Bioglass 45S5 acts as a rigid filler, enhancing the mechanical integrity of the polymer matrix by restricting polymer chain mobility.^30^ However, in TBCHA 30 BG 3, the increase in *E*′ was relatively modest, indicating that at lower TBCHA content, the reinforcement effect of Bioglass was less pronounced. The *E*″ quantifies the viscous response of the material, representing the energy dissipated as heat during cyclic deformation. The data indicate that the TBCHA 50 group exhibited higher *E*″ values than the TBCHA 30 group, reflecting the presence of stronger intermolecular interactions and a more rigid polymer network. However, adding Bioglass slightly reduced *E*″ in both TBCHA 30 and TBCHA 50 groups. This could be attributed to the increased polymer–filler interactions, which limit polymer chain slippage and reduce internal friction, thus lowering the energy dissipation.^31^

**Figure 6:**
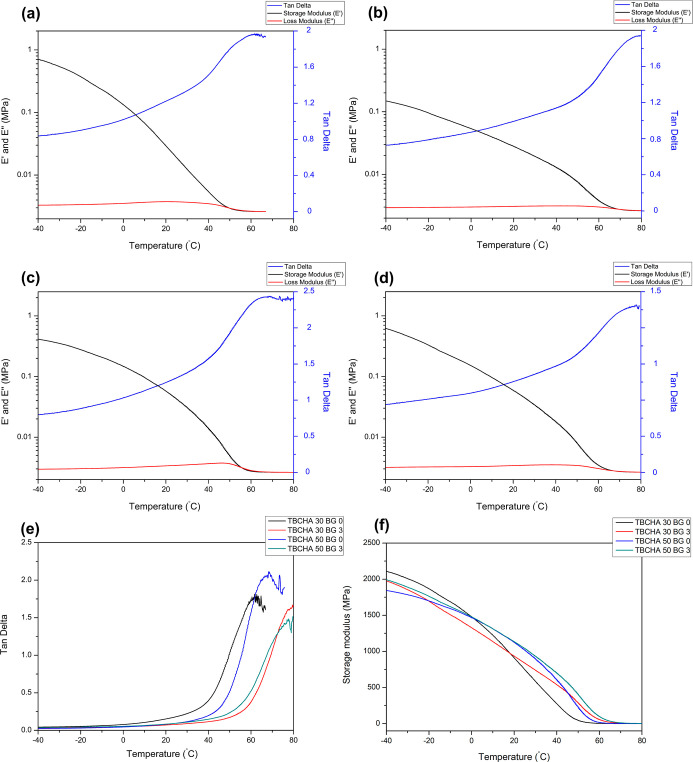
(a) The DMA curves of the TBCHA 30 BG 0 sample; (b) TBCHA 30 BG 3 sample; (c) TBCHA 50 BG 0 sample; (d) TBCHA 50 BG 3 sample; (e) the tan delta of all samples; (f) the storage modulus of all samples.

As illustrated in Figure 6e, tan delta represents the ratio of *E*″ to *E*′, providing insight into the damping behavior and *T*_g_ of the material. The *T*_g_, determined from the peak of the tan delta curve, was higher for the TBCHA 50 group than for the TBCHA 30 group. This increase in *T*_g_ is attributed to the higher cross-link density in TBCHA 50, which restricts molecular mobility and requires a higher temperature for the polymer chains to transition from glass to the rubbery state. Adding 3 wt % Bioglass led to a noticeable shift in *T*_g_, particularly in TBCHA 50 BG 3, where the increase was more pronounced. This can be explained by the strong interactions between the Bioglass particles and the polymer matrix, which further restrict chain mobility. In contrast, for TBCHA 30 BG 3, the change in *T*_g_ was relatively minor, suggesting that Bioglass reinforcement is more effective in highly cross-linked matrices.^30^ It is worth noting that the *T*_g_ values estimated from tan delta peaks in DMA were slightly higher compared to those in the DSC results. This discrepancy is expected due to the different measurement principles; DSC reflects thermal transitions, while DMA detects viscoelastic transitions under mechanical stress. Nevertheless, both results consistently show that *T*_g_ increases with Bioglass incorporation and higher TBCHA content, confirming the thermal stability and shape memory readiness of the SMPC.

Figure 6f illustrates the storage modulus (*E*′) of the TBCHA-based composites as a function of temperature. At 25 °C, the addition of Bioglass 45S5 led to an increase in *E*′ for both TBCHA 30 and TBCHA 50 systems, indicating enhanced stiffness at room temperature. Specifically, *E*′ increased from 745.9 MPa (TBCHA30 BG0) to 837.7 MPa (TBCHA30 BG3), and from 1013.5 MPa (TBCHA50 BG0) to 1040.5 MPa (TBCHA50 BG3). In addition, the TBCHA50 formulations consistently exhibited higher *E*′ than the TBCHA30 formulations, reflecting their intrinsically higher rigidity. Overall, the stiffness trends observed by DMA provide a straightforward basis that is consistent with the flexural-property trends discussed in Figure 7d,e.

**Figure 7:**
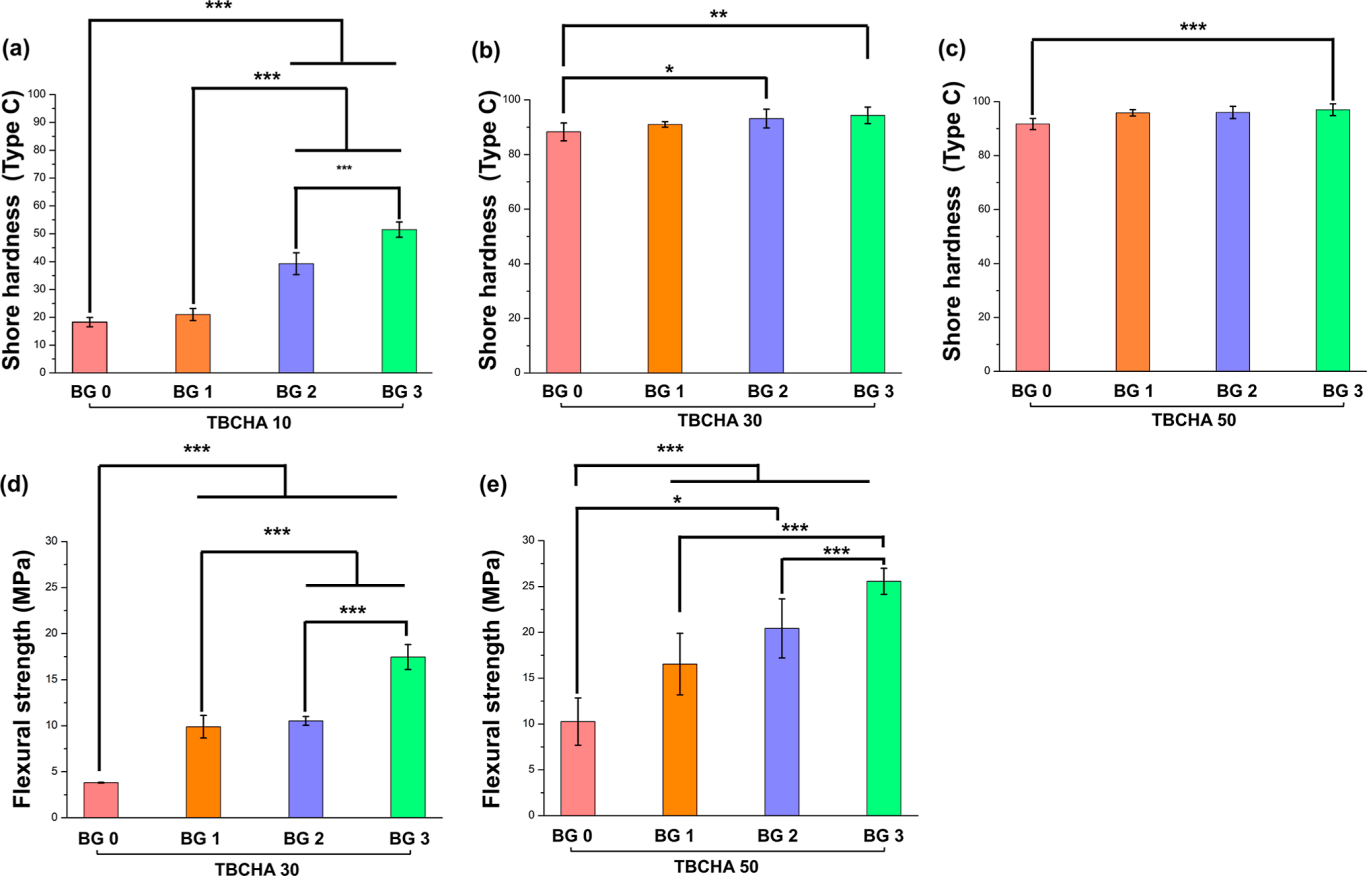
(a) The surface hardness of TBCHA 10 groups; (b) the surface hardness of TBCHA 30 groups; (c) the surface hardness of TBCHA 50 groups; (d) the flexural strength of TBCHA 30 groups (the TBCHA 10 groups could not be measured even with the addition of 3 wt % Bioglass 45S5 due to insufficient strength), and (e) the flexural strength of TBCHA 50 groups. Statistical significance is indicated as *p < 0.05, **p < 0.01, ***p < 0.001. The corresponding ANOVA *R* ^2^ values were 0.965 for (a), 0.444 for (b), 0.566 for (c), 0.996 for (d), and 0.923 for (e).

The Halpin–Tsai equation^32^ was employed to calculate the fitting parameter ξ, which was used to evaluate the interfacial interaction strength between Bioglass 45S5 and the polymer matrix stronger interfacial interaction corresponds to a higher ξ value. The equation is expressed as follows, in eqs 4 and 5

4 $$P/P_{m}=\frac{1+\xi \eta V_{f}}{1-\eta V_{f}}$$

5 $$\eta =\frac{P_{f}/P_{m}-1}{P_{f}/P_{m}+\xi }$$

The *P* denotes the storage modulus of the composite, and *P*_m_ denotes the storage modulus of the neat matrix. *V*_f_ is the volume fraction of Bioglass 45S5 (computed from the weight fraction using the density of 1.28 g/cm^3^ for the matrix, measured by the Archimedes method). In the Halpin–Tsai relation, η accounts for the effect of the filler–matrix property contrast; given the very large modulus contrast between Bioglass (≈60 GPa) and the polymer matrix in DMA, η is expected to be close to unity; therefore, ξ is used as an effective fitting parameter. In this study, we examined the temperature dependence of ξ at 25 and 37 °C; the results are summarized in Table 3. For TBCHA 30 BG 3, ξ = 6.26 at 25 °C and ξ = 52.15 at 37 °C, indicating a pronounced interphase/constraint effect as the temperature approaches *T*_g_. For TBCHA 50 BG 3, ξ is smaller than for TBCHA 30 BG 3, with ξ = 0.815 at 25 °C and ξ = 6.05 at 37 °C, suggesting weaker effective filler–matrix efficiency in this matrix.

**Table 3:** Result of the Fitting Parameter (ξ) of TBCHA 30 BG 3 and TBCHA 50 BG 3

sample	temperature (°C)	*P*/*P*_m_	*V*_f_ (%)	ξ
TBCHA 30 BG 3	25	1.11	1.445	6.264
	37	1.78		52.149
TBCHA 50 BG 3	25	1.03	1.445	0.815
	37	1.1		6.050

These results indicate that relative to TBCHA 50 BG 3, TBCHA 30 BG 3 exhibits stronger interfacial interactions between Bioglass and the matrix, and in both matrices, ξ increases with temperature. This trend arises because the measurements at 37 °C lie closer to each matrix’s *T*_g_ (alpha-transition), where *E*′ is highly sensitive to constrained-chain and interphase effects. The systematically higher ξ in TBCHA 30 BG 3 reflects its lower cross-link density (softer matrix) and lower *T*_g_, which places 37 °C nearer the alpha-transition and enhances interfacial constraint and load transfer relative to TBCHA 50. Therefore, we refer to ξ as an effective ξ that captures interphase and viscoelastic constraints in addition to particle geometry.

This interpretation of ξ is further supported by thermal and spectroscopic analyses. Although DMA and DSC analyses were performed only for the TBCHA 30 and 50 systems due to the poor mechanical integrity of TBCHA 10, the observed *T*_g_ shifts in these compositions are consistent with the interfacial trends revealed by FTIR. The increased content of TBCHA enhances cross-link density through acrylate polymerization, while its bulky cyclohexyl substituents impose additional steric constraints that hinder polymer chain mobility. This steric shielding also reduces the accessibility of polar groups to Bioglass surfaces, indicating that the *T*_g_ elevation arises primarily from physical confinement effects rather than direct interfacial bonding.^15^ Therefore, TBCHA contributes both to an increased cross-link density and to a reduced interfacial interaction efficiency between Bioglass and the surrounding polymer matrix.

### 3.4 Mechanical Properties of TBCHA-Based SMPC

Surface hardness is a critical mechanical property for biomedical implants, directly influencing wear resistance and postimplantation structural support. Figure 7a–c presents the surface hardness test results. The data show that within the same TBCHA monomer samples, an increase in Bioglass 45S5 content corresponds to a rise in hardness. Furthermore, across different TBCHA monomer samples, a higher proportion of TBCHA monomer is associated with an increased hardness.

Figure 7a illustrates the measured hardness values for the TBCHA 10 BG 0, 1, 2, and 3 samples, which were 18.42, 21.00, 39.28, and 51.60, respectively. The hardness values for the TBCHA 30 BG 0, 1, 2, and 3 samples were 88.28, 91.00, 93.16, and 94.30, respectively. Similarly, the TBCHA 50 BG 0, 1, 2, and 3 samples exhibited hardness values of 91.70, 95.30, 96.00, and 97.00, respectively. These results indicate that TBCHA 50 BG 3 demonstrated the highest surface hardness, suggesting superior mechanical performance.

To further investigate the material’s structural support properties and mechanical strength, three-point bending tests were conducted to assess flexural strength. Flexural strength measures a material’s ability to resist deformation and failure under bending loads, reflecting its structural support capability. This study employed the ISO 20795-1 method, classifying denture base polymers and copolymers and defining their requirements. The test specimens were prepared with dimensions of 64 × 3.3 × 10 mm^3^, and the span length was 50 mm.

In the TBCHA 10 groups, the flexural strength could not be measured even with the addition of 3 wt % Bioglass 45S5 due to insufficient strength. Figure 7d,e illustrates the results of the three-point bending tests. Figure 7d presents the flexural strength of the TBCHA 30 groups, with flexural strengths of 4.3, 9.53, 9.9, and 17.46 MPa for TBCHA 30 BG 0, 1, 2, and 3. Figure 7e presents the flexural strength of the TBCHA 50 groups, with flexural strengths of 10.26, 16.54, 20.43, and 25.57 MPa for TBCHA 50 BG 0, 1, 2, and 3. The results indicate that for both the TBCHA 30 and TBCHA 50 groups, the flexural strength of the samples increased as the Bioglass 45S5 content was raised. This enhancement can be attributed to the intact Bioglass 45S5 particles acting as stiffening agents within the matrix. These particles increase the modulus and control deformation under load, allowing the material to distribute stress more efficiently during flexural testing, thereby improving flexural strength.^33^ Regarding the monomer content, samples with higher proportions of the TBCHA monomer exhibited increased flexural strength, following a trend similar to the surface hardness results.

Using the Pukánszky equation,^34^ which considers interfacial interactions and viscoelastic effects, the filler–matrix adhesion in the composites was evaluated. Although the model was originally developed based on tensile strength measurements, flexural strength likewise reflects the load-bearing capability and the efficiency of stress transfer across the interface.^35^ Therefore, this equation was employed to quantify the interfacial interactions in the samples after the incorporation of Bioglass 45S5, as expressed by the following formulas, in eqs 6 and 7

6 $$\sigma_{c}/\sigma_{c}=\frac{1-\phi_{f}}{1+2.5\phi_{f}}\exp\left(B\phi_{f}\right)$$

7 $$\phi_{f}=\frac{w_{f}/\rho_{f}}{w_{f}/\rho_{f}+w_{m}/\rho_{m}}$$

The σ_c_ denotes the flexural strength of the composite, σ_m_ represents the flexural strength of the neat matrix, ϕ_f_ is the volume fraction of Bioglass 45S5, *w*_f_ and *w*_m_ are the weight fractions of the filler (0, 1, 2, and 3 wt %) and matrix (100, 99, 98, 97 wt %), respectively, while ρ_f_ and ρ_m_ correspond to the densities of Bioglass 45S5 (2.7 g/cm^3^) and the matrix (1.28 g/cm^3^, measured using the Archimedes method). The Pukánszky parameter (*B*) serves as a quantitative indicator of filler–matrix interaction.

As summarized in Table 4, its trend is consistent with that obtained from the Halpin–Tsai analysis, indicating that the TBCHA 30 matrix exhibits stronger effective filler–matrix interactions with Bioglass compared to TBCHA 50. Moreover, the calculated *B* values decrease with increasing Bioglass 45S5 content, which can be attributed to an increased probability of particle–particle contacts at higher loadings, promoting filler–filler interactions over filler–matrix interactions.^36^ Nevertheless, the overall flexural strength continues to increase due to the higher rigid-phase fraction and the associated stiffening effect. This trade-off indicates that while absolute strength is enhanced, the interfacial efficiency per unit filler declines, leading to diminishing returns as reflected by the reduction in *B*. Notably, no obvious large agglomerates were observed in the printed composites within the inspected regions (see Supporting Information Section 1.1 SEM/EDS cross-sectional analysis of Bioglass 45S5 dispersion in printed composites), suggesting that the decrease in *B* is mainly associated with increased particle–particle contacts rather than severe macroscopic aggregation.

**Table 4:** Result of the Pukánszky Parameter (*B*) of TBCHA 30 BG 3 and TBCHA 50 BG 3

sample	ϕ_f_ (%)	σ_c/_σ_m_	*B*
TBCHA 30 BG 1	0.477	2.49	120
TBCHA 30 BG 2	0.958	2.59	109
TBCHA 30 BG 3	1.445	4.57	97
TBCHA 50 BG 1	0.477	1.61	103.9
TBCHA 50 BG 2	0.958	1.99	73.5
TBCHA 50 BG 3	1.445	2.49	64

The relative difference in *B* and its decreasing trend with increasing filler content further corroborate the findings from FTIR. The bulky cyclohexyl and *tert*-butyl substituents in TBCHA reduce the accessible interface for hydrogen bonding or ionic coordination, thereby weakening chemical adhesion but promoting a more uniform physical confinement of Bioglass within the matrix.^20^ This confined interphase transfers stress primarily through steric anchoring rather than covalent interaction,^37^ accounting for the gradual hardening but reduced interfacial efficiency observed at high TBCHA ratios. Thus, the mechanical results reinforce the conclusion that TBCHA’s structural rigidity governs a shift from chemical to physical interfacial reinforcement.

### 3.5 Shape Memory Behavior of TBCHA-Based SMPC

Figure 8 illustrates the Rf results. As discussed in the DSC section, the TBCHA 10 groups were not subjected to shape memory evaluation. Under the present bending-based testing protocol, the TBCHA 10 BG 0 sample, with a *T*_g_ below room temperature, and the Bioglass 45S5-containing samples (BG 1 to BG 3) all failed to exhibit any shape memory behavior, as they remained mechanically too soft to sustain bending deformation, even at 3 wt % Bioglass 45S5. Figure 8a shows the Rf result for the TBCHA 30 group; the Rf of the TBCHA 30 BG 0 sample was inferior compared with that of the TBCHA 50 BG 0 sample (Figure 8b). However, upon adding Bioglass 45S5, a notable improvement in the Rf was observed for TBCHA 30 BG 1 and TBCHA 30 BG 2. As explained in the DSC analysis, this improvement is attributed to incorporating Bioglass 45S5 particles, which restrict the mobility of molecular chain segments, enhancing the structural stability of the SMPCs and increasing the sample’s *T*_g_. Nevertheless, the Rf slightly declined when the Bioglass 45S5 content reached 3 wt %. This reduction is primarily due to the excessive addition of Bioglass 45S5, which disrupts the SMPs’ network structure, leading to reduced structural stability and a lower Rf.^38^ However, a statistical analysis revealed that this decrease was not significant.

**Figure 8:**
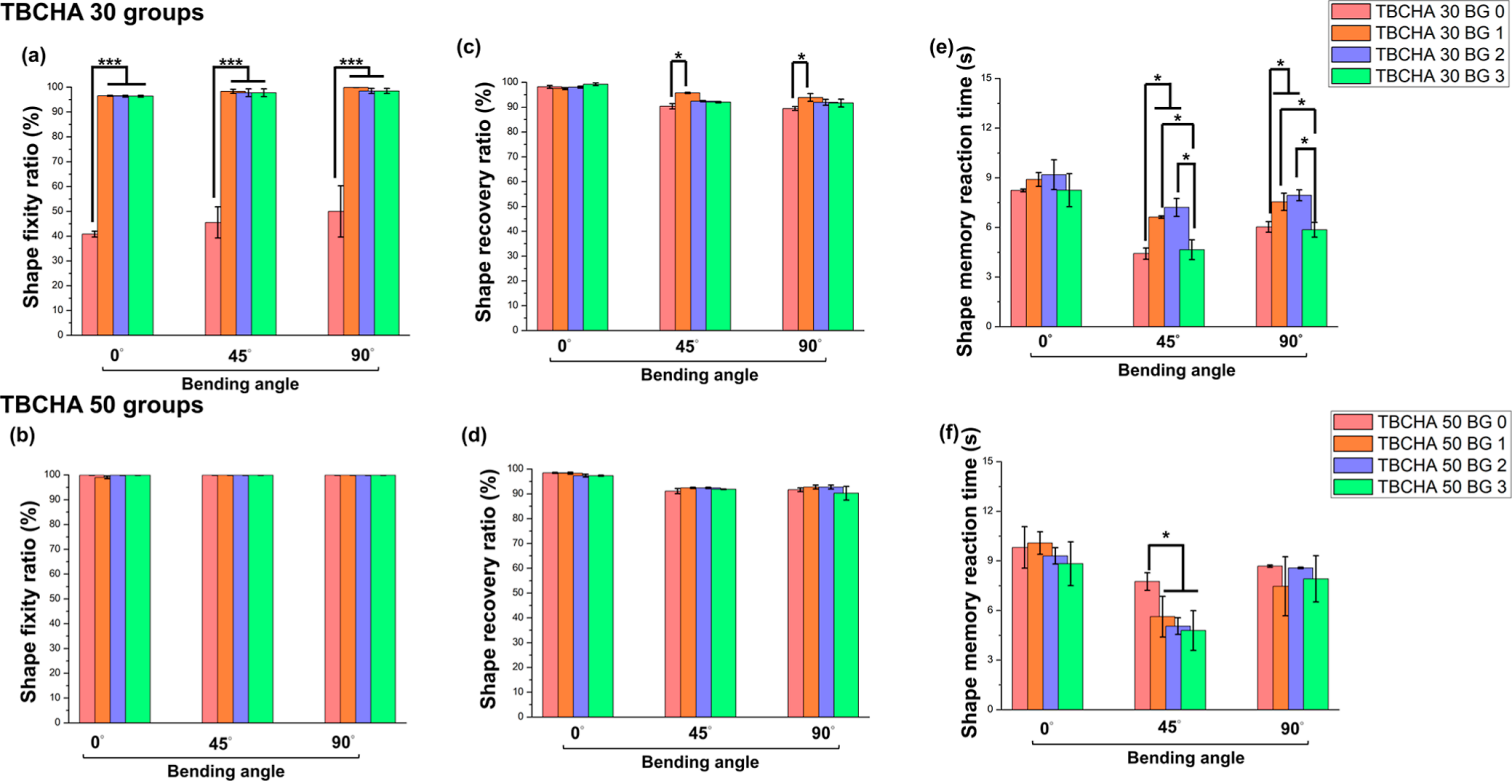
(a) The Rf of TBCHA 30 groups; (b) the Rf of TBCHA 50 groups; (c) the Rr of TBCHA 30 groups; (d) the Rr of TBCHA 50 groups; (e) the Rt of TBCHA 30 groups, and (f) the Rt of TBCHA 50 groups. Statistical significance is indicated as *p < 0.05, **p < 0.01, ***p < 0.001. ANOVA *R* ^2^: (a) 0.970, (b) 0.833, (c) 0.965, (d) 0.948, (e) 0.954, and (f) 0.863.

The observed Rf trends align with the mechanical and viscoelastic characterizations. Hardness and flexural testing demonstrated that Bioglass particles act as rigid fillers, while DMA showed increased *T*_g_ and reduced chain mobility with a higher Bioglass content. Effective interfacial parameters (*B*, ξ) revealed a stronger filler–matrix interaction in TBCHA 30 than in TBCHA 50, but a decrease in efficiency with increasing filler content due to aggregation. This explains the pronounced Rf improvement in TBCHA 30 at low Bioglass 45S5 loadings and the slight reduction at 3 wt %, whereas in TBCHA 50, the high cross-link density and intrinsic rigidity dominated shape fixity, resulting in consistently high Rf (∼100%). These findings align with the changes in tan delta, highlighting a correlation between molecular mobility, filler content, and shape fixity performance.

These macroscopic Rf trends can be rationalized by the interfacial confinement mechanism revealed by FTIR and mechanical modeling. Even without strong chemical interaction, the rigid Bioglass particles embedded in the TBCHA network act as physical anchors, restricting segmental relaxation during cooling.^20^ The cyclohexyl ring’s rigidity complements this confinement, resulting in stable shape fixity despite reduced hydrogen-bonding potential. This physically anchored network explains why the addition of Bioglass 45S5 effectively enhances Rf in TBCHA 30 while maintaining near-perfect fixity in the rigid TBCHA 50 system.

Figure 8c,d illustrates the Rr results. It can be observed that both the TBCHA 30 and TBCHA 50 groups exhibited a similar trend, regardless of the presence of Bioglass 45S5. The shape recovery ratio was the highest when the samples were bent to 0°. This phenomenon is attributed to the greater stored energy modulus of the molecular chains within the SMPCs under greater bending strains. At 0°, the maximum curvature radius facilitates a higher shape recovery ratio.^39^ Furthermore, for both the TBCHA 30 and TBCHA 50 groups, the Rr approached 100% when bent to 0°, with no significant differences observed between the groups. The Rt is the SMP’s duration for completing the shape recovery process after activation. A shorter reaction time indicates a faster and more efficient shape memory response, enabling the SMP to revert to its programmed shape upon prompt stimulation. This property is critical in determining the feasibility of using the material for implant design. Figure 8e,f illustrates the Rt results. The TBCHA 30 and TBCHA 50 groups demonstrated the fastest Rr when bent to 45°, regardless of whether Bioglass 45S5 was added. This is attributed to the fact that the 45° angle corresponds to the smallest radius of curvature during the test, meaning that the material was bent more tightly. This tight bending resulted in stress concentration within the material, making it easier for the stored energy to be released upon stimulation, enabling a quicker return to its original shape.^40^ The results also revealed differences compared to tensile-based shape recovery rate tests. Specifically, samples with higher TBCHA monomer content exhibited variations in the bending shape recovery rates. As discussed in the Rf section, this difference is due to the lower cross-link density in TBCHA 30 samples compared to TBCHA 50 samples. Lower cross-link density allows for greater molecular chain mobility, enabling the chain segments to recover more rapidly to their original configuration under high-temperature conditions.^41^

Figure 9 illustrates the cyclic shape memory performance over five consecutive thermomechanical cycles for TBCHA30 BG 3 and TBCHA50 BG 3. Both formulations exhibited consistently high shape fixity and recovery with only a slight decrease as the cycle number increased. Notably, TBCHA50 BG 3 maintained Rf and Rr values above 99% throughout the five cycles, whereas TBCHA30 BG 3 remained above 97%, demonstrating the robust cyclic stability of the TBCHA-based SMP composites containing Bioglass under the applied programming and recovery conditions.

**Figure 9:**
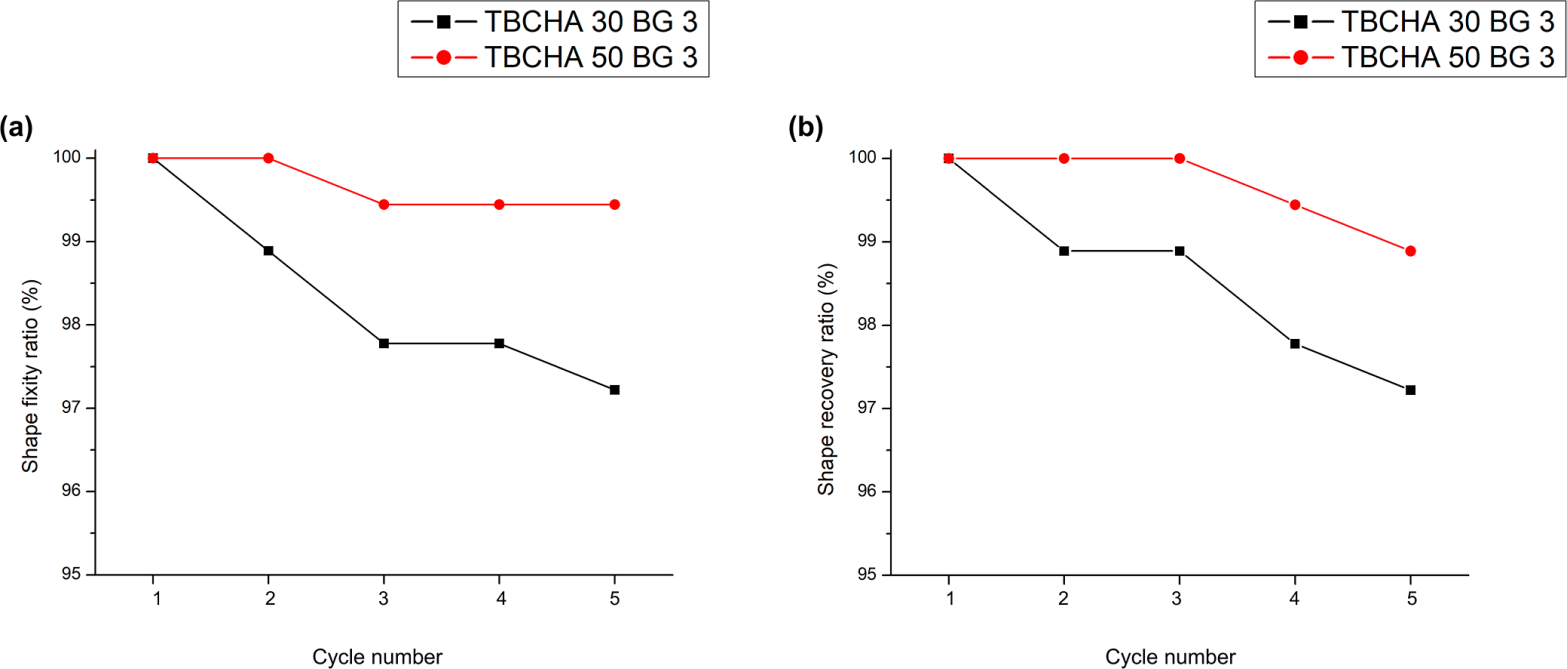
Cyclic shape memory performance over five consecutive thermomechanical cycles: (a) shape fixity ratio (Rf) and (b) shape recovery ratio (Rr) for TBCHA30 BG 3 and TBCHA50 BG 3.

### 3.6 Degradation Properties of TBCHA-Based SMPC

To evaluate the degradation ability of the samples containing PCL-DA and Bioglass 45S5, degradation tests were conducted on the SMPC samples with added Bioglass 45S5. Since Bioglass 45S5 is composed of a mixture of metal and nonmetal oxides, during the degradation process, these ions are released into the degrading solution, increasing the pH. In biological fluids, this localized pH elevation could potentially lead to cell death and implant failure. Therefore, this study measured the pH values of the degradation solutions collected at different time points. Figure 10a–c illustrates that adding Bioglass 45S5 increased the pH of the degradation solution. Furthermore, as the Bioglass 45S5 content increased, the rate of pH elevation gradually slowed. This phenomenon is attributed to the slower degradation rate of Bioglass 45S5, which moderates the pH rise. This characteristic of Bioglass 45S5 has been utilized in drug delivery applications,^42^ providing a potential direction for future research. Considering the long-term implications of pH changes during degradation for practical applications, SMPC containing 3 wt % Bioglass 45S5 was subjected to a one-month extended observation. The results indicated a pH trend of an initial increase followed by a decrease. The initial pH rise is attributed to the degradation of Bioglass 45S5, while the subsequent decline is likely due to the continued degradation of SMP after partial Bioglass 45S5 degradation. As PCL is the primary monomer in the SMP, its degradation produces acidic byproducts such as 6-hydroxycaproic acid, leading to a decrease in the pH of the solution.^43^

**Figure 10:**
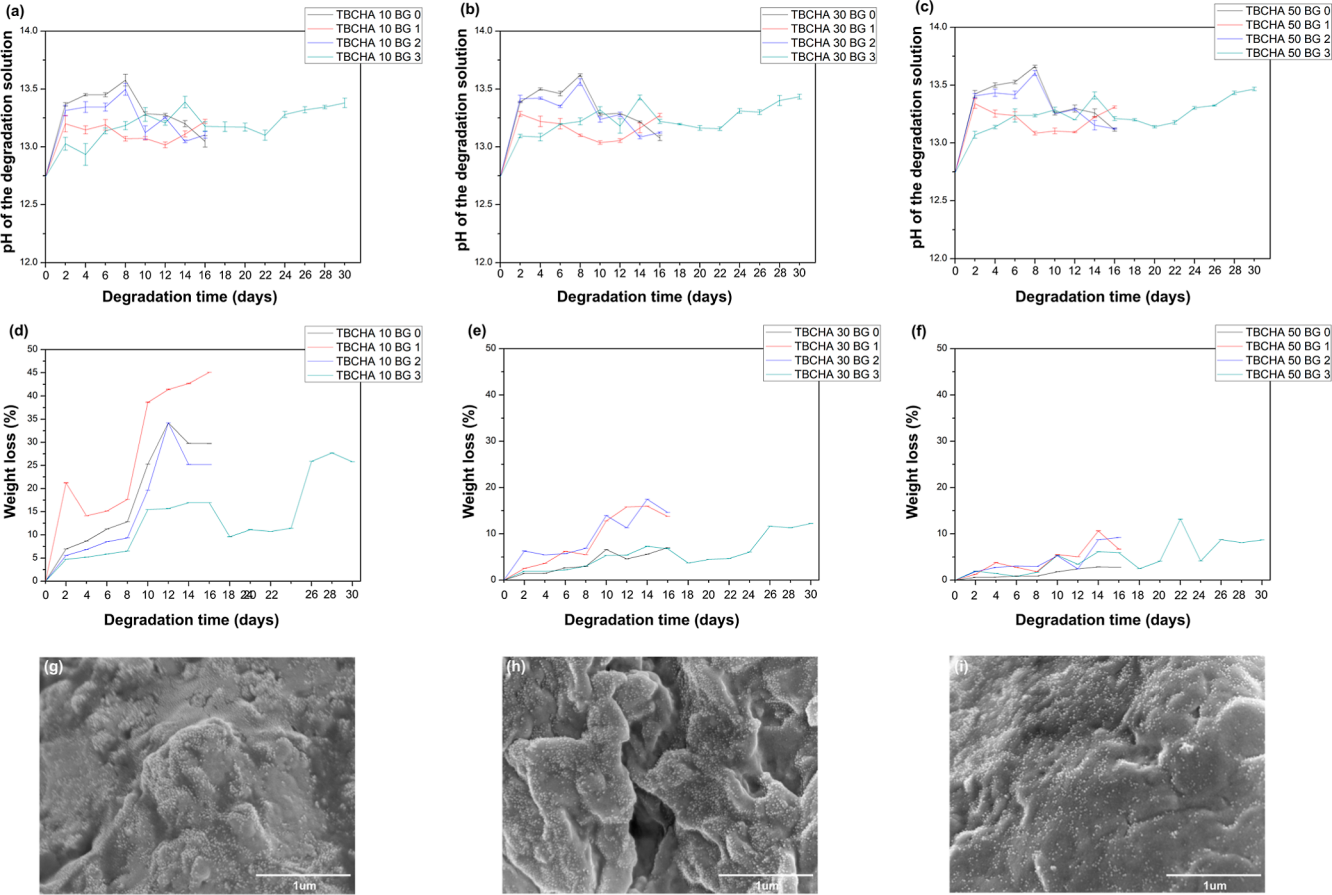
Degrading solution pH value of (a) TBCHA 10 groups; (b) TBCHA 30 groups; (c) TBCHA 50 groups; the weight loss of (d) TBCHA 10 groups; (e) TBCHA 30 groups; (f) TBCHA 50 groups; and the SEM micrographs of (g) TBCHA 10 BG 3 sample (magnification: ×35,000); (h) TBCHA 30 BG 3 sample (magnification: ×35,000); (i) TBCHA 50 BG 3 (magnification: ×35,000) sample after 18 days of immersion in PBS.

Figure 10d–f illustrates the weight loss results of all groups. It was observed that samples with higher PCL-DA monomer content, such as TBCHA 10 groups, and those subjected to longer degradation durations exhibited more significant weight loss, indicating a stronger degradation ability. Regarding adding Bioglass 45S5, incorporating 1 wt % Bioglass 45S5 led to an increase in weight loss, primarily due to the enhanced hydrophilicity of the SMPs facilitated by the appropriate amount of Bioglass 45S5.^44^ However, when the Bioglass 45S5 content was increased to 2 wt %, a decrease in degradation rate was observed. This reduction can be attributed to the higher Bioglass 45S5 content, which reduced the hydrophilicity of the samples and, as Bioglass 45S5 degrades more slowly than SMPs, resulted in lower weight loss.^45^ This observation parallels the trends seen in the mechanical and shape memory analyses. While the addition of Bioglass 45S5 enhances rigidity and restricts chain mobility, excessive loading reduces interfacial efficiency and creates more tortuous diffusion pathways, thereby hindering solution penetration. For SMPCs with 3 wt % Bioglass 45S5, the weight loss exhibited a distinct trend compared to previous samples, with a decrease in weight loss at specific time points. This phenomenon is likely due to HA forming on the surface of Bioglass 45S5 after prolonged immersion in the degradation solution. Given that PBS is a phosphate-buffered solution, the alkaline environment facilitates HA formation, increasing sample weight.^46^ SEM and EDS analyses were conducted to verify this hypothesis.

Subsequent morphology analysis was conducted on samples from each group containing 3 wt % Bioglass 45S5 after 18 days of degradation. Figure 10g–i illustrates the surface morphology of the Bioglass 45S5 in the TBCHA-based SMP, which was examined. The results revealed that after 18 days of immersion in the degrading solution, a layer of fine particulate deposits had formed on the surface of Bioglass 45S5. Table 5 shows the EDS analysis, which indicates the presence of calcium (Ca) and phosphorus (P) elements in the TBCHA 10 BG 3 sample, suggesting that during degradation, Bioglass 45S5 reacted with the solution to form Ca and P deposits, thereby contributing to the observed weight increase. In contrast, only Ca was detected for the TBCHA 30 BG 3 and TBCHA 50 BG 3 samples. This phenomenon is primarily attributed to the increased cross-link density within SMPC as the TBCHA monomer content increased. The enhanced cross-link density hinders the penetration of the degrading solution into the molecular chains of the SMPCs, leading to a reduced degradation rate.^47^ Consequently, the reaction rate of Bioglass 45S5 is slower. Moreover, Ca deposition during Ca and P layer formation occurs before P deposition,^48^ explaining the absence of P in the EDS spectra of the TBCHA 30 BG 3 and TBCHA 50 BG 3 samples after 18 days of degradation.

**Table 5:** EDS analysis of TBCHA 10 BG 3, TBCHA 30 BG 3, and TBCHA 50 BG 3 after 18 Days of Immersion in PBS

	after 18 days of immersion in PBS		
element	TBCHA 10 BG 3	TBCHA 30 BG 3	TBCHA 50 BG 3
Si	3.9%	16.4%	18.8%
Ca	69.6%	32.1%	13.5%
Na	16.8%	51.4%	67.6%
P	9.7%	0%	0%

The degradation behavior exhibited a clear composition- and filler-dependent trend. At low Bioglass 45S5 contents (≤1 wt %), the degradation rate increased due to the enhanced hydrophilicity and localized ion exchange introduced by the glass surface, which promoted PBS solution infiltration and ester bond cleavage within the PCL-DA segments. However, at higher Bioglass 45S5 loadings (≥2 wt %), this trend reversed: the degradation rate slowed markedly because the denser Bioglass framework created a continuous rigid phase that obstructed diffusion pathways and limited solution penetration into the polymer network.^45^ The hydrophobic and sterically bulky cyclohexyl and *tert*-butyl groups in TBCHA further modulate this effect by reducing the surface polarity of the matrix and minimizing water accessibility at the Bioglass–polymer interface. This reduced wettability minimizes hydrolytic attack and stabilizes the filler–matrix boundary. The same nonpolar shielding that suppresses hydrogen bonding in FTIR now contributes to enhanced hydrolytic stability. Overall, the interaction between Bioglass and the sterically shielded matrix leads to a dual functionality: at low loadings, it accelerates degradation by increasing interfacial wettability; at higher loadings, it physically reinforces the network and restricts diffusion. Although the cyclohexyl structure sterically limits hydrogen bonding with Bioglass, this trade-off yields a more stable interfacial microenvironment, mitigating particle detachment and hydrolytic degradation commonly observed in conventional polyester-based SMPCs.

The experimental results further revealed that the overall degradation rate increased with rising TBCHA content and Bioglass 45S5 concentration. Among the samples with 3 wt % Bioglass 45S5, TBCHA 30 BG 3 exhibited a balanced degradation rate, making it appropriate for bone and cartilage scaffolds, whereas TBCHA 50 BG 3 showed the slowest degradation rate, highlighting its potential for long-term or load-bearing implant applications.

Additional experiments were performed in noncatalyzed PBS to gain deeper insight into the in vivo degradation behavior of TBCHA-based SMPC (see Supporting Information, Section 1.6: The degradation test of the TBCHA-based SMPC). Remarkably, the pH of the immersion medium remained stable at approximately 7.0 throughout 30 days, indicating a minimal release of acidic or alkaline byproducts. This pH stability is favorable for maintaining a physiological microenvironment and reducing the risk of cytotoxic responses.

To estimate the potential in vivo degradation profile, a degradation acceleration factor was derived by comparing the weight loss data obtained under alkaline-catalyzed and noncatalyzed conditions. The time required for 50% material degradation under physiological conditions was projected by extrapolating the catalyzed degradation data using this factor. This approach facilitates the establishment of a practical degradation time frame, supporting the rational design of TBCHA-based SMPC for long-term biomedical applications. The degradation behavior of SMPCs is a crucial factor influencing their biomedical applications, particularly in tissue engineering and temporary implant systems. Table 6 presents the time required to reach 50% degradation. It can be observed that as the cross-linking density increases, the degradation time is prolonged. Specifically, TBCHA 10 BG 3, TBCHA 30 BG 3, and TBCHA 50 BG 3 require approximately 1.75, 3.7, and 6.3 years to reach 50% degradation under noncatalyzed conditions. In contrast, the degradation timelines of clinically available biodegradable implants are well documented. Inion CPS fixation systems, widely used in cranio-maxillofacial surgery, are reported to undergo complete bioresorption within 2 to 4 years (pediatric versions: 2 to 3 years).^49^ Similarly, PLLA/PGA copolymer applications such as LactoSorb or RapidSorb retain sufficient mechanical strength for 6 to 8 weeks, with complete resorption occurring by 12 to 18 months.^50^ Pure PLLA implants exhibit even slower degradation, with in vitro degradation taking about 2 years, and clinical settings reporting persistence of more than 3.5 years.^51^ In guided bone regeneration, resorbable collagen membranes such as Geistlich Bio-Gide typically provide barrier function for 4 to 8 weeks, followed by complete resorption within a few months.^52^ Other membranes like Ossix Plus have been observed to resorb by around 8 months in vivo.^53^ While data on specific bone graft substitutes (e.g., β-TCP, calcium sulfate, HA) vary, their clinical resorption spans range from 1 month to several years, depending on material type. These established clinical timeframes highlight the balance between early mechanical support and gradual resorption needed for effective bone healing.

**Table 6:** Time Required to Reach 50% Degradation of TBCHA 10 BG 3, TBCHA 30 BG 3, and TBCHA 50 BG in Non-catalyzed PBS

sample	*t*_50_ (years): time to 50% degradation
TBCHA 10 BG 3	1.75
TBCHA 30 BG 3	3.74
TBCHA 50 BG 3	6.32

The 0.5 to 1% monthly weight loss observed in noncatalyzed PBS suggests that the TBCHA-based SMPC would reach approximately 50% degradation within 2 to 6 years. This time frame is comparable to the degradation profile of long-term resorbable implants such as PLLA-based fixation devices or Inion CPS systems, which are designed to provide mechanical support for several years before complete resorption. Such degradation behavior indicates that the TBCHA-based SMPC is particularly suitable for applications requiring prolonged stability, including dental screws or load-bearing scaffolds, where gradual resorption is advantageous for balancing long-term support with eventual replacement by host tissue.

### 3.7 Shape Memory Behavior after Degradation Properties of TBCHA-Based SMPC

To evaluate the degradation behavior of SMPC samples containing PCL-DA and Bioglass 45S5, degradation tests were performed on specimens with varying Bioglass 45S5 contents, as shown in Section 3.4 (Properties of Degradation), and the impact of Bioglass 45S5 incorporation on sample degradation was assessed. Furthermore, the effect of degradation on shape memory properties was investigated. Figure 11a–c illustrates the results for the TBCHA 30 BG 0 and TBCHA 30 BG 3 samples after 7 and 14 days of degradation. The findings indicate that Rr, Rt, and Rf remained unchanged compared to the undegraded samples. These results demonstrate that after 14 days of accelerated degradation, the samples maintained excellent shape memory performance. Similarly, Figure 11d–f illustrates the results for the TBCHA 50 BG 0 and TBCHA 50 BG 3 samples after 7 and 14 days of degradation. A comparable trend was observed with Rr, Rt, and Rf remaining consistent with the undegraded samples. This confirms that the samples retained superior shape memory properties after 14 days of accelerated degradation. Overall, the results suggest that increasing the TBCHA or Bioglass 45S5 content enhances the degradation lifespan while effectively preserving the shape memory performance of the TBCHA-based SMPC.

**Figure 11:**
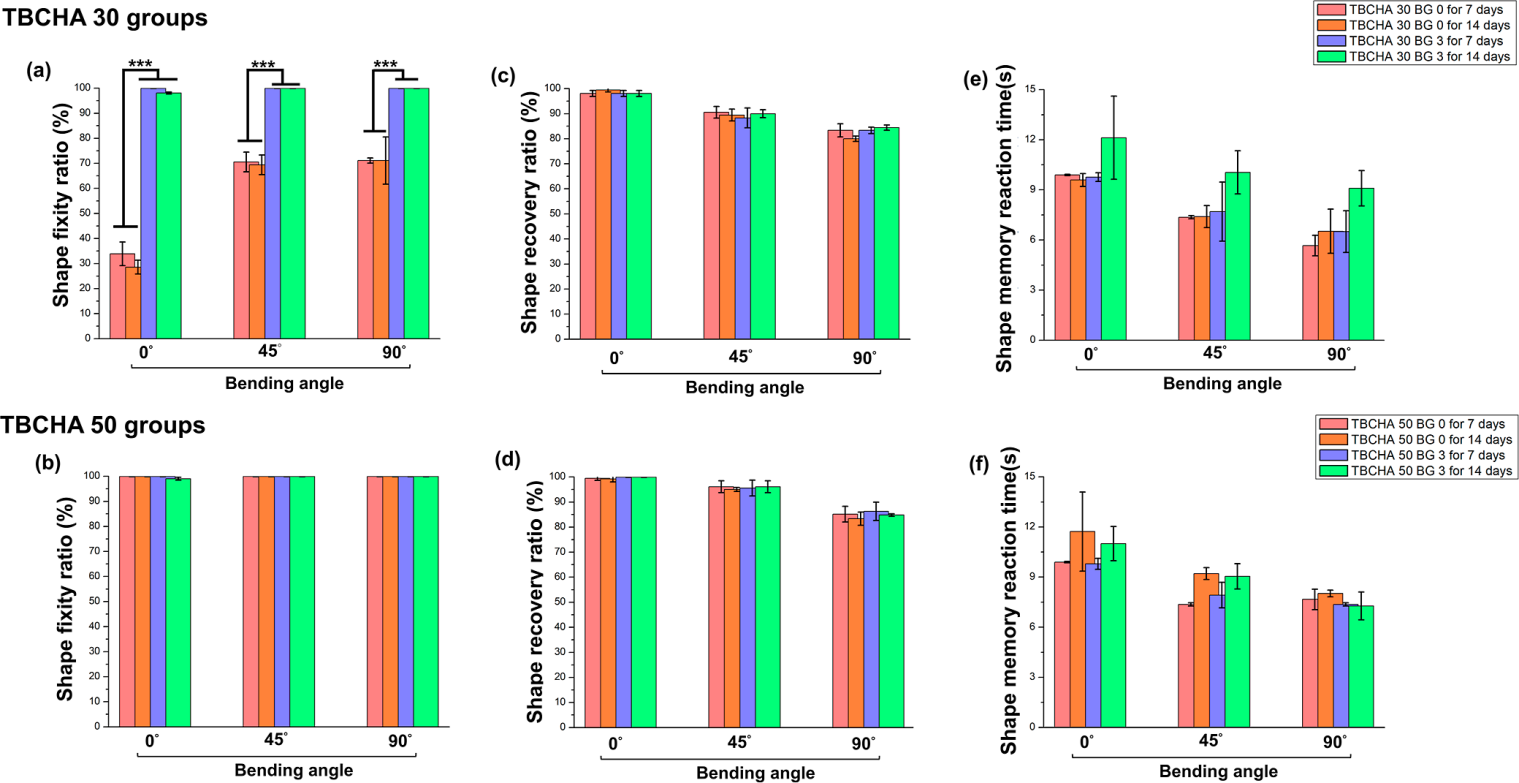
(a) The Rf of TBCHA 30 groups after 7 and 14 days of degradation (the red bar is bending to 0°; the blue bar is bending to 45°; the green bar is bending to 90°; the green bar is added 3 wt % Bioglass 45S5); (b) the Rt of TBCHA 30 samples after 7 and 14 days of degradation; (c) the Rr of TBCHA 30 samples after 7 and 14 days of degradation; (d) the Rf of TBCHA 50 samples after 7 and 14 days of degradation; (e) the Rt of TBCHA 50 samples after 7 and 14 days of degradation and (f) the Rr of TBCHA 50 groups after 7 and 14 days of degradation. Statistical significance is indicated as *p < 0.05, **p < 0.01, ***p < 0.001. ANOVA *R* ^2^: (a) 0.820, (b) 0.821, (c) 0.825, (d) 0.946, (e) 0.823, and (f) 0.823.

Compared with current reports on biomedical SMPCs based on PCL matrices or incorporating Bioglass, the present system demonstrates comparable or even superior shape memory performance under degradation conditions. For instance, electrospun triethoxysilane-terminated PCL mats reinforced with Bioglass exhibited excellent performance, with Rf values consistently reaching 100% and Rr values ranging from 92% to 96%,^54^ while electrospun epoxy/PCL nanoweb blends achieved Rf values of 95 to 99% and Rr values between 88% and 100%.^55^ More recently, cross-linked PCL-DA/Bioglass composite scaffolds have been developed as self-fitting implants for repairing complex bone defects, demonstrating the ability to preserve shape memory behavior in clinically relevant architectures.^56^ In comparison, the TBCHA-based PCL-DA/Bioglass 45S5 composites developed in this work not only reached a similar level of Rf and Rr but also maintained these properties after 14 days of accelerated degradation, thereby advancing the state of the art by demonstrating the long-term stability of shape memory functionality under physiologically relevant conditions.

### 3.8 In Vitro Bioactivity of TBCHA-Based SMPC

Based on the experiments mentioned above, it was confirmed that SMP with 3 wt % Bioglass 45S5 exhibits superior shape memory properties, mechanical performance, and thermal stability. Therefore, TBCHA-based SMP with 3 wt % Bioglass 45S5 was explicitly selected from each group for further in vitro bioactivity testing. Figure 12a presents the SEM analyses performed on the control group before immersion in SBF. The surface appeared smooth without any granular deposits, indicating the absence of HA formation at this stage. This control group is a reference for comparison to samples subjected to SBF immersion. The in vitro bioactivity test was conducted to simulate the human physiological environment and assess the formation of HA layers on Bioglass 45S5 within the SMPC. Figure 12b–d illustrates the results of SEM observations, revealing distinct deposition morphologies on the sample surfaces with varying PCL contents. In the case of TBCHA10 BG 3, the lower cross-linking density facilitated ion diffusion from the SBF, promoting reactions with the embedded Bioglass. As a result, a continuous and dense layer of deposits formed on the TBCHA10 BG 3 surface after 28 days of immersion. In contrast, due to the higher cross-linking densities of the TBCHA30 BG 3 and TBCHA50 BG 3 samples, ion diffusion from the SBF was more limited, thereby hindering effective interaction with the Bioglass within the matrix. Consequently, the surfaces of TBCHA30 BG 3 and TBCHA50 BG 3 exhibited sparsely distributed spherical deposits, characteristic of the initial stages of HA formation. This difference in the deposition morphology may thus be attributed to restricted ion transport, resulting in slower HA nucleation and growth.

**Figure 12:**
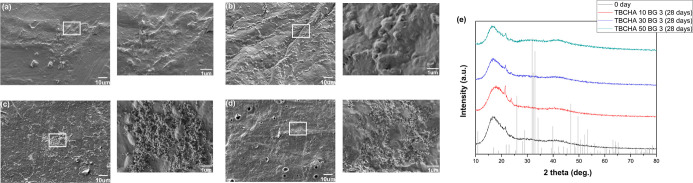
SEM micrographs of (a) TBCHA-based SMP adds 3 wt % Bioglass 45S5 after 0 days of immersion in SBF (magnification (main/inset): ×1000/×5000); (b) TBCHA 10 BG 3 after 28 days of immersion in SBF (magnification (main/inset): ×1000/×15,000); (c) TBCHA 30 BG 3 after 28 days of immersion in SBF (magnification (main/inset): ×1000/×15,000); (d) TBCHA 50 BG 3 after 28 days of immersion in SBF (magnification (main/inset): ×1000/×15,000); (e) XRD of all groups.

Table 7 shows the EDS analysis results. The original sample exhibited a Ca/P ratio of 3.18. After immersion in SBF, the three groups showed varying Ca/P ratios. TBCHA 10 BG 3 displayed a relatively high Ca/P ratio of 2.19, which may be attributed to its low cross-linking density, leading to faster degradation in the SBF environment. This accelerated degradation likely hindered HA formation, suggesting that the material had not yet reached a stable mineralized state (theoretical Ca/P = 1.67).^57^ In contrast, TBCHA 50 BG 3 exhibited a lower Ca/P ratio, possibly due to its higher cross-linking density, which limited the interaction between the embedded Bioglass 45S5 and the SBF, thereby reducing ion release and subsequent deposition. TBCHA 30 BG 3, with a moderate cross-linking density, regulated ion release kinetics more effectively, resulting in a Ca/P ratio closer to the theoretical value of 1.67 (measured at 1.47). This indicates a more favorable environment for the nucleation and growth of apatite-like phases, suggesting comparatively higher bioactivity among the three groups. The difference in the HA deposition across the series mirrors the FTIR-derived interfacial mechanism. The TBCHA 10 matrix, with minimal steric hindrance, permits greater ion diffusion and interfacial exchange with Bioglass, leading to dense HA deposition. Conversely, the rigid, hydrophobic cyclohexyl network in TBCHA 50 limits ion mobility and Bioglass exposure, producing sparse deposits despite identical filler loading.

**Table 7:** EDS Result of TBCHA 10 BG 3, TBCHA 30 BG 3, and TBCHA 50 BG 3 after Immersion in SBF

element	after 0 days of immersion in SBF	after 28 days of immersion in SBF		
samp name	TBCHA-based SMP adds 3 wt % Bioglass 45S5	TBCHA 10 BG 3	TBCHA 30 BG 3	TBCHA 50 BG 3
Si	26.4%	22.8%	16.9%	13.5%
Ca	50.0%	55.8%	42.0%	45.3%
Na	11.5%	1.2%	18.3%	3.4%
P	12.2%	20.2%	22.8%	37.7%
Ca/P ratio	3.18	2.19	1.47	0.9

Since EDS is a semiquantitative technique and the Bioglass 45S5 content was limited to only 3 wt %, further analysis was conducted using XRD to confirm HA formation from a crystallographic perspective. Figure 12e illustrates the XRD result, where the gray light is the HA peak from the JCPDS card, although the broad amorphous hump characteristic of the polymer matrix still dominates the diffraction patterns. For the unimmersion samples (0 days), only the amorphous background of the polymer was observed, with no discernible reflections. After 28 days of SBF immersion, all three groups exhibited weak shoulder peaks within the 31° to 34° range. Among them, the TBCHA 30 BG 3 displayed approximately four weak but distinguishable reflections (at 31.4°, 32.3°, 33.2°, and 34.5°), which correspond to the (211), (112), and (300) planes of HA, 2 theta range (31.7°, 32.9°, and 34.0°). Overall, these results suggest the onset of HA crystallization in all groups, with more pronounced features in TBCHA 30 BG 3, consistent with its Ca/P ratio approaching the theoretical value of 1.67 (Supporting Information, Section 1.7, The XRD peak of the TBCHA-based SMPC, where the XRD peak is presented along with an enlarged comparative diffraction pattern). These findings are consistent with the SEM observations and demonstrate that TBCHA-based SMPC possesses excellent in vitro bioactivity, as evidenced by its ability to induce calcium phosphate deposition on the surface. To decouple the intrinsic bioactivity of the glass from matrix effects, a standalone Bioglass 45S5 (without polymer) was also immersed in SBF as a positive control. As expected, abundant “cauliflower-like” apatite formed within 28 days (Supporting Information, Section 1.3, In vitro bioactivity of Bioglass 45S5). Together with weak yet discernible HA reflections at 31 to 34° (2 theta) for TBCHA30 BG3, this control confirms that the limited deposition observed on SMPC surfaces arises primarily from restricted ion transport within the cross-linked polymer network, rather than insufficient bioactivity of the glass phase. This provides promising preliminary evidence supporting their potential as bone regeneration materials.

### 3.9 Results Overview with Proof-of-Concept Bone Screw Fabrication

For a better indication of the overall TBCHA-based SMPC performance, the material properties of all of the samples related to quantitative experimental results are additionally presented in a radar chart in Figure 13. These systems can adjust the composition to optimize mechanical behavior, degradation time, or shape memory properties according to specific application requirements. This chart demonstrates that incorporating Bioglass 45S5 or increasing the cross-link density of the samples can effectively enhance mechanical behavior and shape memory properties while also allowing for control modulation of the degradation rate. Furthermore, to directly visualize the shape memory behavior of the TBCHA-based SMPC, a porous spherical structure was 3D printed through the TBCHA 30 BG 3 and subjected to thermal deformation and recovery cycles. The supplementary video (Supporting Information, Video 1) demonstrates the reversible deformation of the printed structure upon heating above *T*_g_ and its recovery to the original geometry upon reheating, thereby confirming both the printability and the intrinsic shape memory capability of the developed resin system.

**Figure 13:**
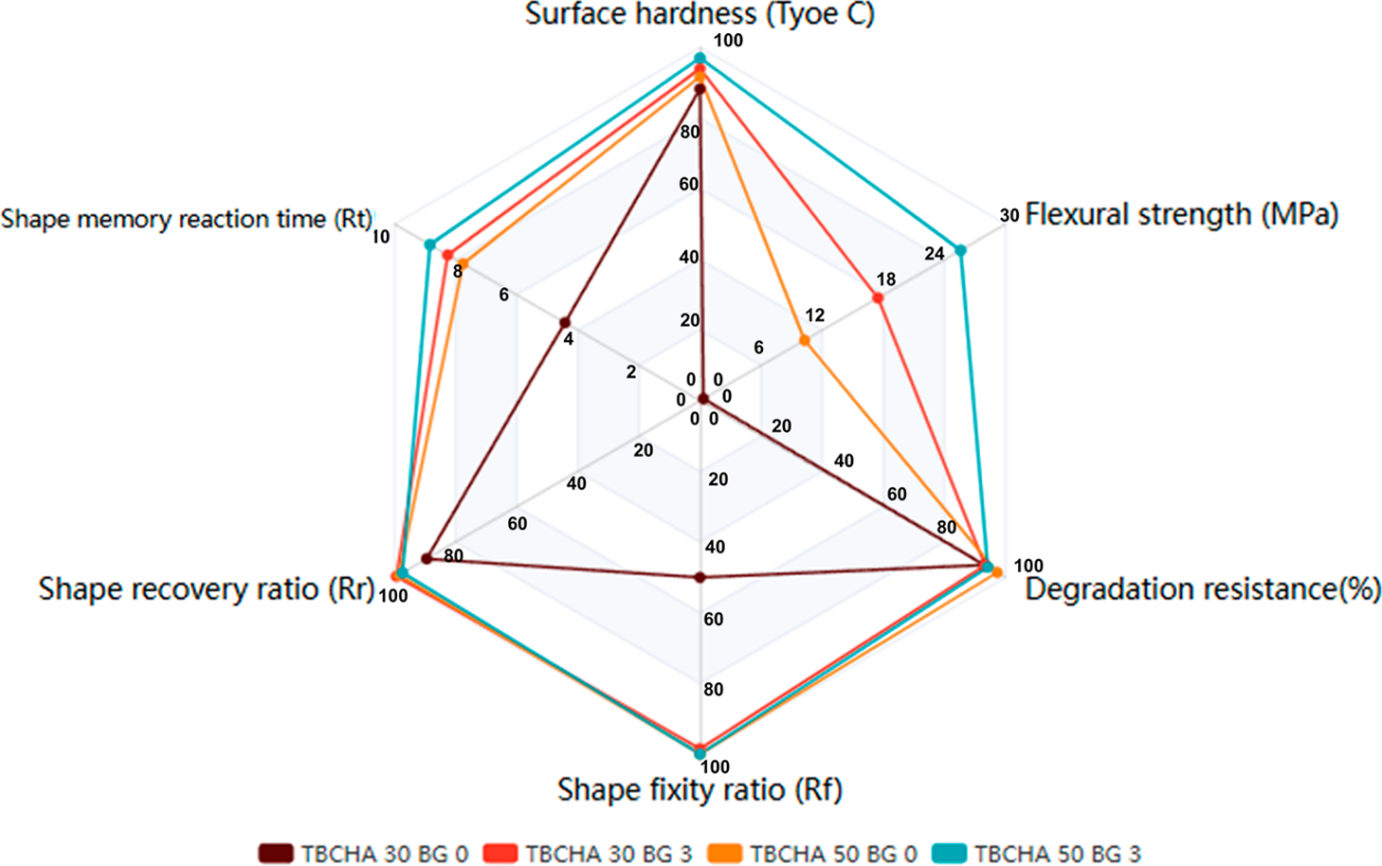
Overall performance with the TBCHA-based SMPC related to the material testing results.

To verify the potential applicability of the developed material in biomedical fixation, a bone screw was 3D printed using TBCHA 30 BG 3, based on an ISO 5835 shallow-thread screw model obtained from Thingiverse (Thing: 4030004, scaled for demonstration). Figure 14a shows the original geometry of the 4D-printed bone screw prototype. The printed screw exhibited pronounced thermally induced shape memory behavior: when heated above its *T*_g_, it became soft and could be temporarily deformed into a reduced-diameter configuration, enabling insertion into an undersized predrilled hole, as shown in Figure 14b. Upon reheating, the screw recovered its original geometry (Figure 14c), thereby achieving a secure mechanical fixation within the cavity. This proof-of-concept highlights the potential of the developed SMPC for minimally invasive orthopedic applications.

**Figure 14:**
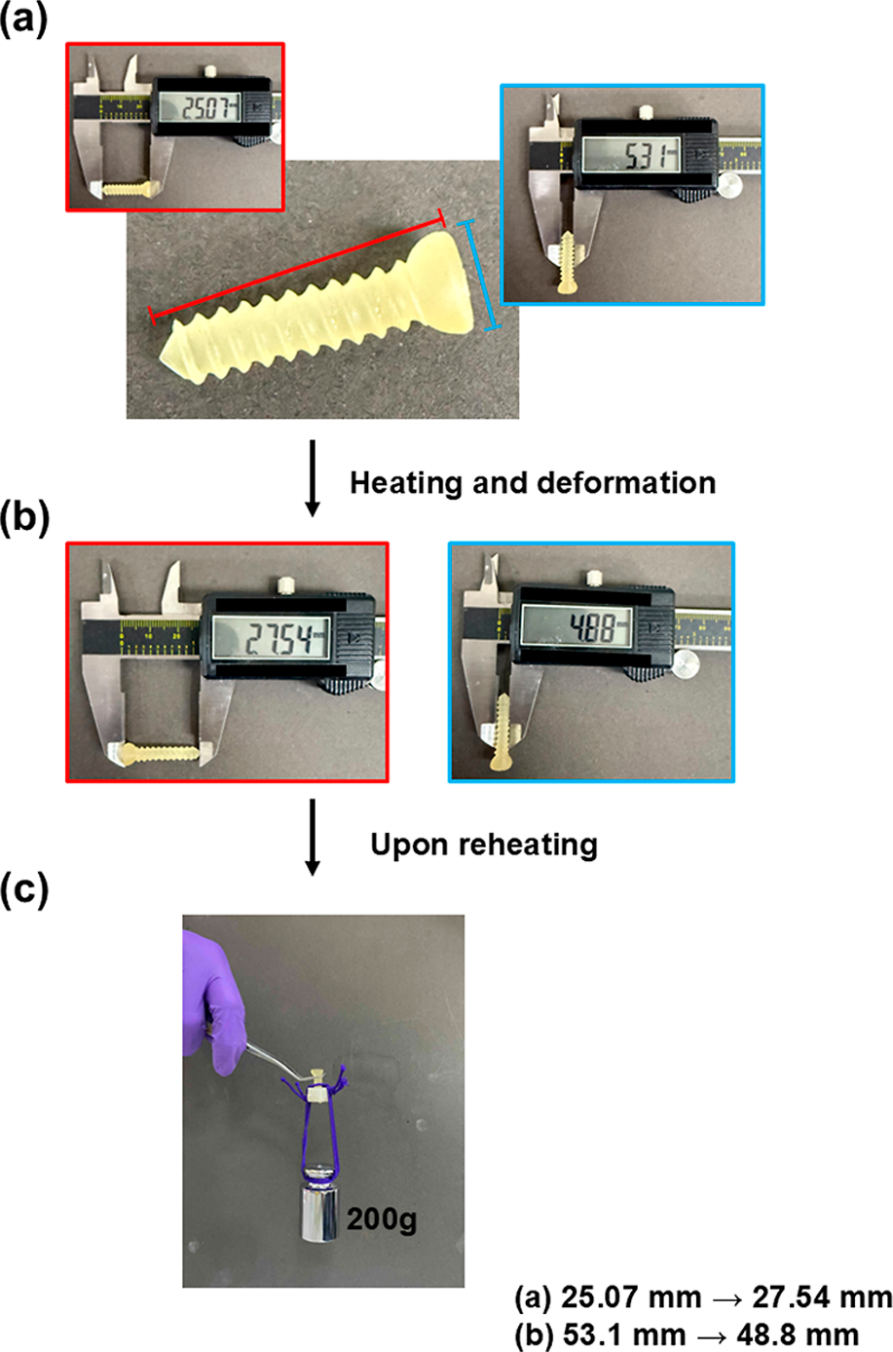
Bone screw printed using TBCHA 30 BG 3: (a) original shape; (b) after heating, the screw can be compressed to a smaller size; and (c) upon reheating, it can be secured within the hole.

## 4 Conclusion

This study successfully developed a 4D printing SMPC by incorporating Bioglass 45S5 into a PCL-DA/TBCHA matrix. Increasing the Bioglass content significantly influenced the thermal stability, mechanical properties, degradation behavior, and shape memory performance, enabling property tailoring for targeted biomedical applications. Beyond compositional optimization, the study elucidated the structural role of TBCHA in governing filler–matrix interactions. The bulky and nonpolar cyclohexyl and *tert*-butyl substituents in TBCHA sterically shield reactive carbonyl and ether groups, reducing hydrogen bonding and Ca^2+^ coordination with Bioglass. This steric confinement shifts the interfacial mechanism from chemical interaction to physical anchoring, promoting interfacial stability and moderating hydrolytic degradation at high cross-link densities. Consequently, TBCHA acts as a molecular regulator that balances the rigidity, interfacial adhesion, and hydrophobic protection. Together, these insights establish a material design framework in which cross-link density and filler loading can be strategically tuned to achieve long-term mechanical robustness, shape memory reliability, and bioactive functionality in next-generation biodegradable 4D-printed implants. This interfacial regulation strategy could be extended to other hydrophobic monomers or filler systems, providing a generalizable framework for designing stable, bioactive 4D-printable SMP composites.

## Supplementary Material





## Data Availability

During the preparation of this work, the author(s) used ChatGPT (OpenAI) and Grammarly (Grammarly Inc.) in order to improve the English language. After using these tools, the author(s) reviewed and edited the content as needed and take full responsibility for the content of the publication. All data necessary to support the conclusions of this study are included in the article and its Supporting Information.

## Notes

Supplementary SEM/EDS analysis, printing-parameter and degree-of-conversion evaluation, in vitro bioactivity results, ANOVA *R* ^2^ values, particle-size analysis, degradation data, and supplementary XRD results (PDF)
Reversible deformation of the printed structure upon heating above *T*_g_ and its recovery to the original geometry upon reheating (MP4)
