# Supplementary material for: 4D Printing of TBCHA-Based Shape Memory Polymer Composites: Bioglass 45S5 Addition Improves Stability Retention and Shape Memory Performance
Source: ACS Omega. 2026 Apr 29;11(18):27063–82. doi: 10.1021/acsomega.6c00690 (PMC13177222; doi:10.1021/acsomega.6c00690)
Supplement: Supplementary file 1 [file ao6c00690_si_001.pdf]

4D printing of TBCHA-based shape memory polymer composites: Bioglass 45S5 addition improves stability retention and shape memory performance

Meng-Ruei Liu [a], Hsuan Chen [b], Nien-Ti Tsou [a], Shyh-Yuan Lee [b,c], Yuan-Min Lin [b,c,*]

[a] *Department of Materials Science and Engineering, National Yang Ming Chiao Tung University, Hsinchu, 30010, Taiwan*

[b] *Department of Dentistry, National Yang Ming Chiao Tung University, Taipei, 112304, Taiwan*

[c] *Institute of Oral Tissue Engineering and Biomaterials, National Yang Ming Chiao Tung University, Taipei, 112304, Taiwan*

* Email: ymlin@nycu.edu.tw

Supplementary Material

1.1 SEM/EDS cross-sectional analysis of Bioglass 45S5 dispersion in printed composites

Figure S1 Representative SEM images and EDS elemental maps of the printed composites containing Bioglass 45S5: (a) TBCHA30 BG 3 and (b) TBCHA50 BG 3. The fracture surfaces show no obvious large agglomerates or sedimentation-induced concentration gradients within the inspected regions. The corresponding EDS maps of the characteristic Bioglass elements (e.g., Ca, Si, and Na) exhibit dispersed distributions across the analyzed areas, supporting homogeneous microscale dispersion after printing.

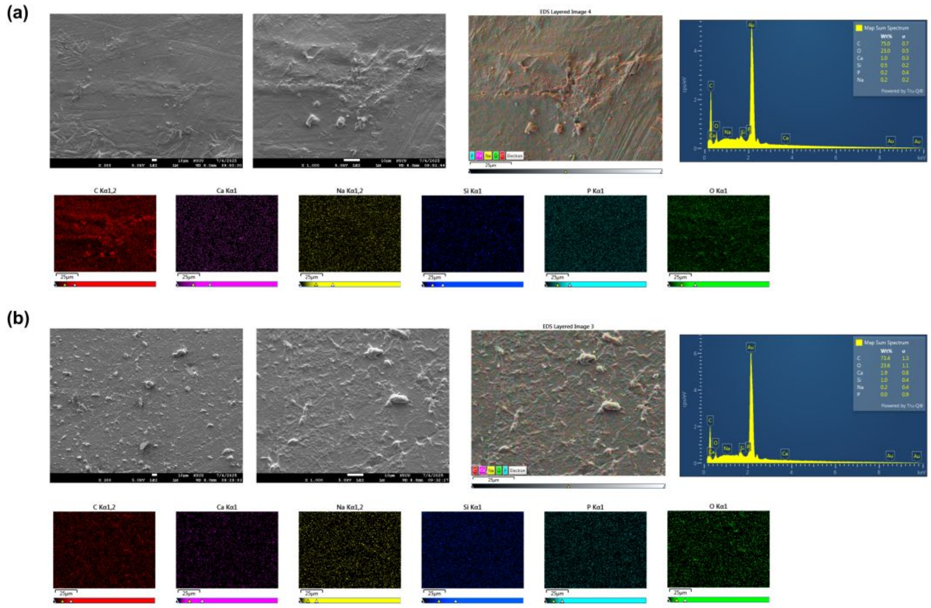

Figure S1 Representative SEM images, EDS elemental maps, and EDS spectra of the Bioglass 45S5-filled printed composites: (a) TBCHA30 BG 3 (Magnification

(main/inset): ×300 / ×1,000) and (b) TBCHA 50 BG 3 (Magnification (main/inset): ×300 / ×1,000).

1.2 Printing parameters test

Because incorporating Bioglass particles can reduce UV light penetration and thereby alter the curing behavior of the photocurable resin, exposure-time and degree-of-conversion (DC) tests were performed on the formulations containing the highest Bioglass content, namely TBCHA30 BG 3 and TBCHA50 BG 3. Table S1 summarizes the exposure-time screening results. After 45 s of UV curing, the cured thickness of both formulations approached ~0.10 mm. Therefore, an exposure time of 45 s per layer and a layer thickness of 0.10 mm were used for printing.

Figure S2 shows the FTIR spectra of the uncured resin and the printed specimens after 15 min of post-curing. The DC was calculated by monitoring the decrease of the alkenyl band (C=C) at 1638 cm^{-1} relative to the carbonyl band (C=O) at 1720 cm^{-1}, using the following DC Eqs.1:

$$Degree\ of\ conversion\ (\%) = 1 - \frac{Cured\ [\ ^{C=C}/_{C=O}\ ]}{Uncured\ [\ ^{C=C}/_{C=O}\ ]} \times 100\% \qquad \text{Eqs.1}$$

As summarized in Table S2, after printing with an exposure time of 45 s per layer followed by 15 min of post-curing, the calculated DC values were 80.6% (TBCHA30 BG 3) and 78.4% (TBCHA50 BG 3). These values fall within the typical conversion

range reported for photopolymerization-based additive manufacturing, confirming the feasibility of the selected printing parameters.

Table S1 Exposure time of TBCHA 30 BG 3 and TBCHA 50 BG 3.

Sample name	Cure-time (s)	Cure-depth (mm)
TBCHA 30 BG 3	15	0.04
	30	0.07
	45	0.12
	60	0.25
TBCHA 50 BG 3	15	0.03
	30	0.05
	45	0.11
	60	0.2

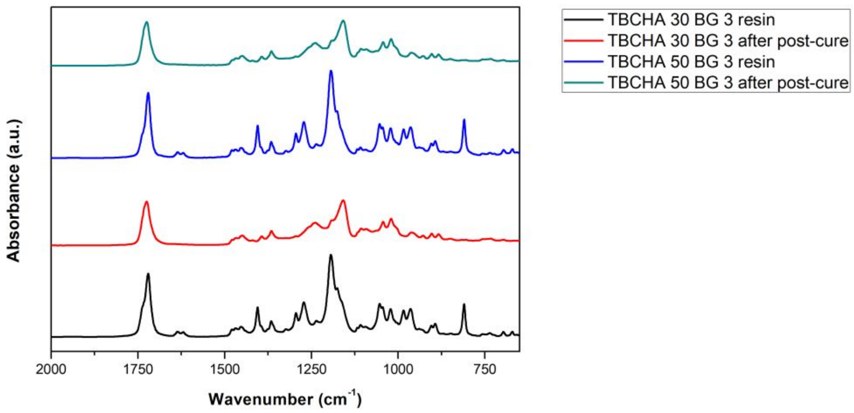

Figure S2 The FTIR spectrum of TBCHA 30 BG 3 and TBCHA 50 BG 3 before and after post-cure.

Table S2 The degree of conversion of TBCHA 30 BG 3 and TBCHA 50 BG 3.

Sample name	Degree of conversion (%)
**TBCHA 30 BG 3**	**80.6%**

**TBCHA 50 BG 3**	**78.4%**

1.3 In vitro bioactivity of Bioglass 45S5

Figure S3 (a) to (h) illustrates the SEM observations, revealing a clear time-dependent evolution of the Bioglass 45S5 surface during immersion in SBF. At 0 days, the surface appeared smooth and free of deposits. After 1 day, small spherical particles emerged, marking the onset of calcium phosphate nucleation. Over 3 and 5 days, these particles grew, became denser, and progressively covered the surface, forming a rough and porous layer. At 7 days, a continuous network-like structure was observed, indicating the development of a coherent Ca-P layer. With further immersion for 14 and 21 days, the network became thicker and more uniform, while at 28 days, the surface was fully covered by a dense and homogeneous layer, characteristic of a mature hydroxyapatite coating.

Table S3 shows the EDS analysis. A noticeable trend was observed with prolonged immersion: starting from day 5, the calcium (Ca) peaks began to increase. This phenomenon can be attributed to the rapid release of Na^+ and Ca^{2+} from Bioglass 45S5 during the initial stage of SBF immersion, leading to a localized increase in Ca^{2+} concentration. This promotes the early formation of a calcium-phosphate (Ca-P) layer,

specifically a hydroxycarbonate apatite (HA) phase, which initially forms a highly enriched surface layer. After day 7, the deposited Ca-P layer gradually transforms into a more stable hydroxyapatite (HA) phase, progressing from the outermost surface inward. At this stage, a densely packed HA layer likely forms on the surface, reducing further deposition of Ca and P on the external layer. As a result, EDS measurements show a decline in surface Ca content. Consistent with this, SEM morphological analysis reveals the formation of a dense-like layered structure on the surface of Bioglass 45S5, further confirming the progressive transformation of the Ca-P layer into a compact HA coating [1].

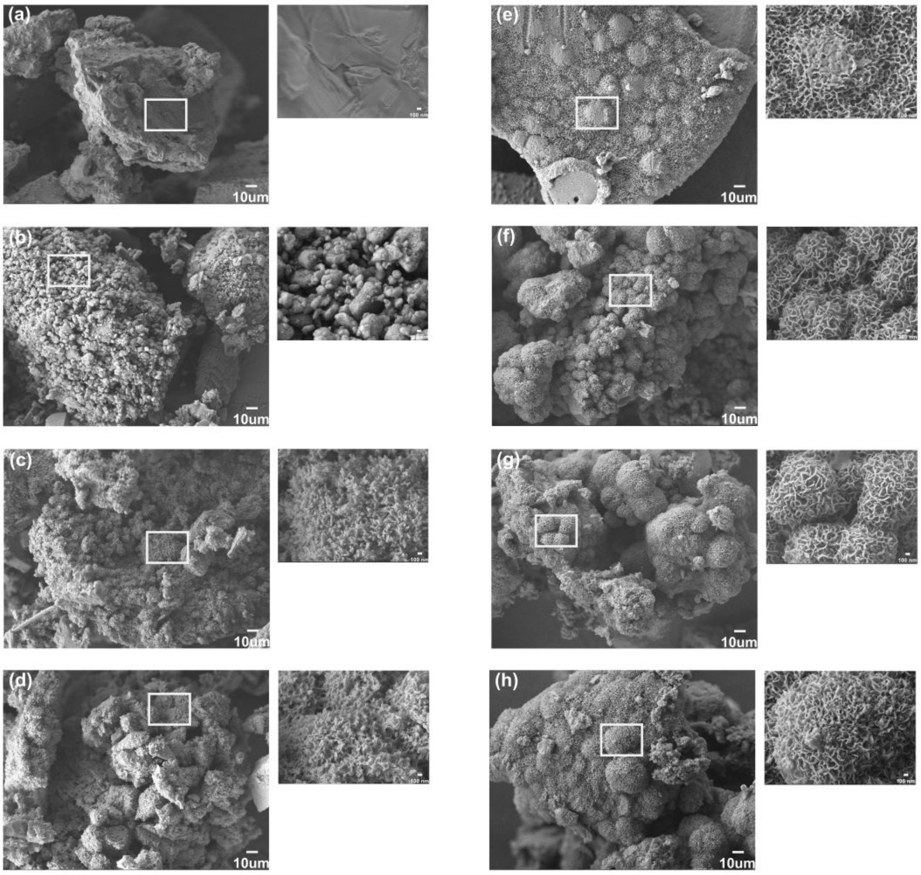

Figure S3 SEM micrographs of the sample surfaces after (a) 0, (b) 1, (c) 3, (d) 5, (e) 7, (f) 14, (g) 21, and (h) 28 days of SBF immersion (main images: ×5,000; inset images: ×35,000).

Table S3 The Bioglass 45S5 EDS analysis result after immersion in SBF.

**Sample**	**0 days**	**1 day**	**3 days**	**5 days**	**7 days**	**14 days**	**21 days**	**28 days**
Si	40.3	55.5	46.5	3.6	53.9	30.4	12	1.8
Ca	30.7	20.9	32.1	86.5	53.9	42.3	53.6	56.5
Na	24.6	22.2	14.4	2.2	1.4	3	2.1	8.1
P	4.3	1.3	7	7.7	30.6	24.3	32.2	33.7

1.4 Statistical analysis (ANOVA R^2 values)

Note: For shape-memory metrics, the ANOVA R^2 values are reported as before degradation / after degradation, where black indicates before degradation and red indicates after degradation.

Table S4 The ANOVA R^2 values of all test

Test	Group name	ANOVA R^2 values
Shore hardness	TBCHA 10	0.965
Shore hardness	TBCHA 30	0.444
Flexural strength		0.996
Shape fixity ratio		0.97/0.82
Shape recovery ratio		0.965/0.825
Shape recovery time		0.954/0.823
Shore hardness	TBCHA 50	0.566
Flexural strength		0.923
Shape fixity ratio		0.833/0.821
Shape recovery ratio		0.948/0.946
Shape recovery time		0.863/0.823

1.5 Particle size analysis

The particle size distribution of melt-quenching Bioglass 45S5 was quantified from SEM micrographs using ImageJ. Three representative SEM images acquired at the magnifications shown in Figure S4 were used for the analysis. Prior to measurement, the ImageJ scale was calibrated based on the scale bar in each micrograph. Individual particles were measured manually using the line tool in ImageJ to obtain the particle length and width (i.e., the maximum and minimum diameters), while overlapped or

severely agglomerated regions were excluded from the analysis. In total, 90 particles (*n* = 90) were analyzed across the three images, and the results are reported as the particle-size distribution in the main text.

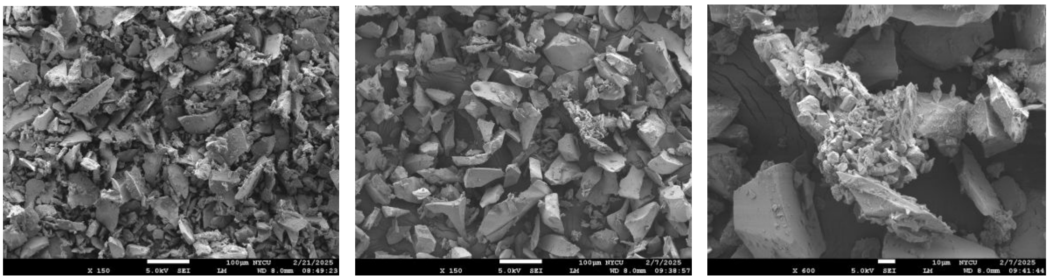

Figure S4 Representative SEM micrographs of melt-quenching Bioglass 45S5 particles used for particle-size analysis. Particle dimensions were measured in ImageJ using the line tool (maximum and minimum Feret diameters), excluding overlapped or severely agglomerated regions. Magnifications and scale bars: (a) 150×, 100 μm; (b) 150×, 100 μm; (c) 600×, 10 μm.

1.6 The degradation test of the TBCHA-based SMPC

To further investigate the in vivo degradation behavior of the TBCHA-based SMPC, all samples containing 3 wt% Bioglass 45S5 were immersed in non-alkaline phosphate-buffered saline (PBS). Figure S5 (a) illustrates the pH variation of the degradation solution over 30 days. The results show that the pH remained stable at around seven throughout the immersion duration, indicating that the degradation

process did not induce significant alkalinity or acidity. This suggests that the material is unlikely to cause adverse local pH shifts in vivo, thereby minimizing the risk of cell death and implantation failure due to pH-related cytotoxicity. Figure S5 (b) illustrates the weight change of the samples during the 30 days immersion. The observed trend was similar to that of the samples degraded under alkaline catalysis, wherein the samples initially lost mass but gradually exhibited an increase in weight after day 18. This phenomenon is attributed to the deposition of calcium and phosphorus compounds (such as hydroxyapatite precursors) on the surface of Bioglass 45S5. The degradation rates of catalyzed and non-catalyzed samples were compared to estimate the long-term durability of the SMPCs under physiological conditions. The time required to reach 50% weight loss (t_{50}) was selected as a representative indicator of functional degradation (i.e., potential loss of shape memory performance). Based on the extrapolated calculations, the degradation behavior of the specimens was quantitatively assessed using weight loss measurements. Specifically, t_{50}% was defined as the time required for the samples to reach 50% weight loss, serving as an indicator of overall degradation kinetics. In addition, W_6 and W_{12} represent the weight loss determined after 6 and 12 days of immersion, respectively. These parameters were employed to evaluate and compare the degradation profiles of the investigated systems. The degradation time was estimated using the following Eqs.2:

$$t_{50}\% = \frac{50}{W_{12} - W_6 / 12 - 6} \div 365$$ Eqs.2

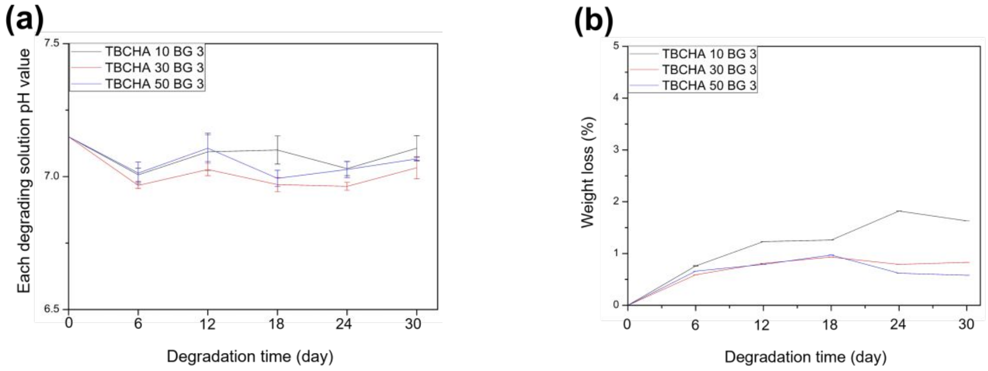

Figure S5 The degrading solution pH value of (a) adding 3wt% BG; the weight loss of (b) adding 3wt% BG.

1.7 The XRD peak of the TBCHA-based SMPC

Figure S6 illustrates the XRD results of before immersion in the SBF sample (0 day), (b) TBCHA 10 BG 3, (c) TBCHA 30 BG 3, and (d) TBCHA 50 BG 3 after 28 days of immersion. To better resolve the weak reflections of hydroxyapatite, the XRD patterns were re-plotted by restricting the 2 theta range to 30° to 35°. In addition, the curves were smoothed using Origin software to minimize background noise without altering peak positions. This treatment facilitated clearer identification of shoulder peaks at 31° to 34°. Peak positions were identified using baseline subtraction followed by local maxima detection within the 30° to 35° range to ensure objective and consistent determination

across all groups.

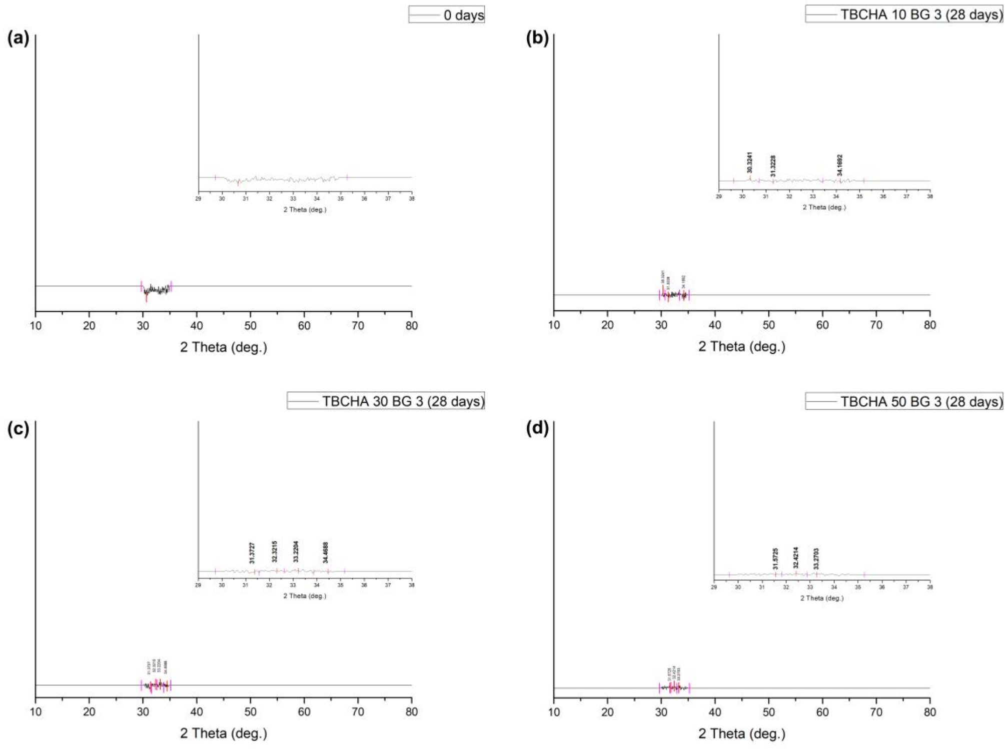

Figure S6 The XRD results of (a) before immersion in the SBF sample (0 day), (b) TBCHA 10 BG 3, (c) TBCHA 30 BG 3, and (d) TBCHA 50 BG 3 after 28 days of immersion.

References

1. Cerruti, M., D. Greenspan, and K. Powers, *Effect of pH and ionic strength on the reactivity of Bioglass 45S5.* Biomaterials, 2005. **26**(14): p. 1665–1674.
